# Nuclear translocation of spike mRNA and protein is a novel feature of SARS-CoV-2

**DOI:** 10.3389/fmicb.2023.1073789

**Published:** 2023-01-26

**Authors:** Sarah Sattar, Juraj Kabat, Kailey Jerome, Friederike Feldmann, Kristina Bailey, Masfique Mehedi

**Affiliations:** ^1^Department of Biomedical Sciences, University of North Dakota School of Medicine and Health Sciences, Grand Forks, ND, United States; ^2^Biological Imaging Section, Research Technology Branch, National Institute of Allergy and Infectious Diseases, National Institutes of Health, Bethesda, MD, United States; ^3^Division of Intramural Research, National Institute of Allergy and Infectious Diseases, National Institutes of Health, Hamilton, MT, United States; ^4^Department of Internal Medicine, Pulmonary, Critical Care, and Sleep and Allergy, University of Nebraska Medical Center, Omaha, NE, United States

**Keywords:** SARS-CoV-2, spike, NLS, nuclear translocation, mRNA

## Abstract

Severe acute respiratory syndrome coronavirus 2 (SARS-CoV-2) causes severe pathophysiology in vulnerable older populations and appears to be highly pathogenic and more transmissible than other coronaviruses. The spike (S) protein appears to be a major pathogenic factor that contributes to the unique pathogenesis of SARS-CoV-2. Although the S protein is a surface transmembrane type 1 glycoprotein, it has been predicted to be translocated into the nucleus due to the novel nuclear localization signal (NLS) “PRRARSV,” which is absent from the S protein of other coronaviruses. Indeed, S proteins translocate into the nucleus in SARS-CoV-2-infected cells. S mRNAs also translocate into the nucleus. S mRNA colocalizes with S protein, aiding the nuclear translocation of S mRNA. While nuclear translocation of nucleoprotein (N) has been shown in many coronaviruses, the nuclear translocation of both S mRNA and S protein reveals a novel feature of SARS-CoV-2.

## Introduction

The recently emerged severe acute respiratory syndrome coronavirus 2 (SARS-CoV-2), along with SARS-CoV and Middle East respiratory syndrome coronavirus (MERS-CoV), belong to the *Coronaviridae* virus family. The current ongoing outbreak has shown that SARS-CoV-2 is highly pathogenic and more transmissible than SARS-CoV or MERS-CoV (Liu et al., 2020). These coronaviruses contain a positive-strand RNA genome with a few unique features: two-thirds of the viral RNA is translated into a large polyprotein, and the remainder of the viral genome is transcribed by a discontinuous transcription process into a nested set of subgenomic mRNAs (Pasternak et al., 2006; Perlman and Netland, 2009; Sola et al., 2015). The different subgenomic RNAs encode four conserved structural proteins (spike, S; envelope, E; membrane, M, and nucleocapsid, N) and several accessory proteins (Bojkova et al., 2020; Finkel et al., 2021). The S protein of both SARS-CoV and SARS-CoV-2 interacts with the host cell receptor angiotensin converting enzyme 2 (ACE2) and triggers fusion between the viral envelope and host cell membrane to facilitate successful viral entry (Jia et al., 2005; Hoffmann et al., 2020). However, the S protein of MERS-CoV binds to dipentidyl peptidase (DPP4) to facilitate entry into cells (Raj et al., 2013). Importantly, the SARS-CoV-2 S protein is a significant pathogenic factor because of its broad tropism for mammalian ACE2 (Conceicao et al., 2020). While the S protein is an attractive target for therapeutic development (Martinez-Flores et al., 2021), the lack of comprehensive information on S protein expression and subcellular translocation hinders the identification of an effective S protein-targeting therapeutic to combat SARS-CoV-2 infection.

The genome sequence is generally the blueprint for detecting biological function (Harrow et al., 2009). Thus, the S protein’s function is encoded in the S gene sequence. Identifying novel features in the S gene sequence, its expression and subcellular localization may shed light on the unique pathogenesis of SARS-CoV-2 compared to other pathogenic beta-coronaviruses, particularly SARS-CoV and MERS-CoV. A recent study showed several SARS-CoV-2 genomic features, including novel sequence insertions and enhanced N protein nuclear localization signals (NLSs) that are thought to be responsible for the unique pathogenesis of this coronavirus (Gussow et al., 2020). There are three types of NLSs: pat4, pat7, and bipartite. The pat4 signal is a chain of 4 basic amino acids consisting of lysine or arginine or three basic amino acids, with the last amino acid being either histidine or proline. The pat7 signal begins with proline and is followed by six amino acids, which contains a four-residue sequence in which three of the four residues are basic. The bipartite signal consists of two basic amino acids with a 10-residue spacer and a five amino acid sequence in which at least three of the five amino acids are basic (Robbins et al., 1991; Hicks and Raikhel, 1995; Rowland et al., 2005). The subcellular localization of some SARS-CoV-2 proteins has been studied *in vitro* (Zhang J. et al., 2020), but a comprehensive understanding of the subcellular localization of the S protein is missing.

Here, we first report the nuclear translocation of S protein and mRNA in SARS-CoV-2-infected cells. The translocation of the SARS-CoV-2 S mRNA appeared to be assisted by the S protein, which contains an NLS motif that is unique among human pathogenic beta-coronaviruses.

## Results

### The novel NLS motif “PRRARSV” is in the S protein of SARS-CoV-2 but not SARS-CoV or MERS-CoV

**Figure 1:**
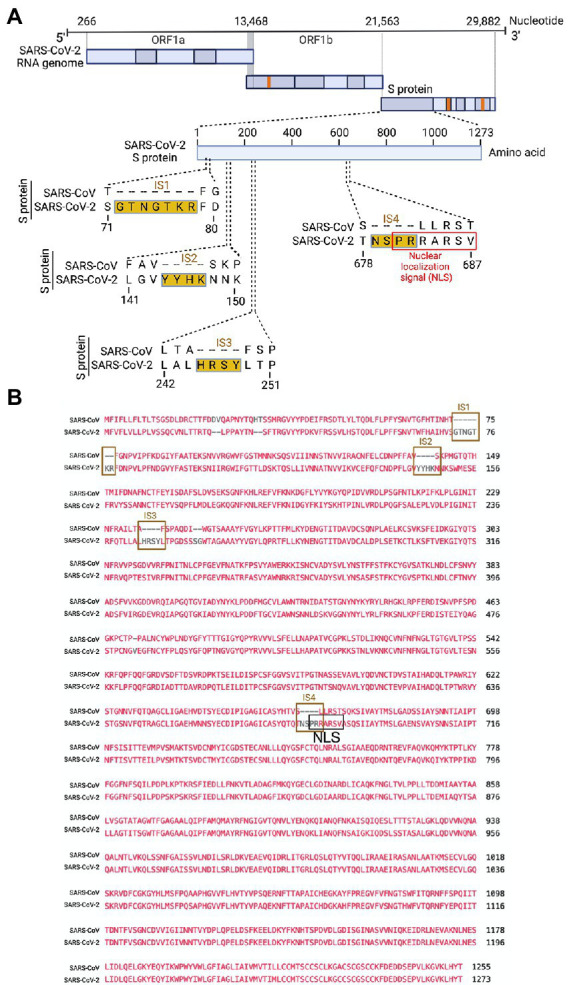
Only the SARS-CoV-2 S protein had an NLS motif “PRRARSV” due to a novel sequence insertion. **(A)** Full-length SARS-CoV-2 genome (nucleotide) (USA/WA-CDC-WA1/2020 isolate, GenBank accession no. MN985325) and open reading frames (ORF) are shown at the top. The SARS-CoV-2 S protein amino acid sequence was aligned with SARS-CoV (Urbani strain, GenBank accession no. AY278741) by NCBI’s constraint-based multiple alignment tool COBALT (Papadopoulos and Agarwala, 2007), and the relative positions of four novel sequence insertions (ISs) are shown in the S protein ORF as follows: IS1: “GTNGKTR,” IS2: “YYHK,” IS3: “HRSY,” and IS4: “NSPR.” The fourth IS (NSPR) created a pat7 NLS “PRRARSV” in the S protein (shown in the red rectangle). **(B)** The S protein ORF sequences between SARS-CoV-2 and SARS-CoV were aligned, and the Lined rectangles highlight the four novel insertions: IS1, IS2, IS3, and IS4. The IS4 “NSPR” created a pat7 NLS “PRRARSV” in the S protein (shown in the black rectangle).

Several groups have reported novel nucleotide insertions in the S gene of SARS-CoV-2, as indicated by a multiple sequence alignment for the S protein sequences of different coronaviruses, such as a polybasic site “PRRA” produced by a 12-nucleotide acquisition at the S1-S2 boundary through multiple host-species adaptations (Andersen et al., 2020; Walls et al., 2020). However, S protein sequence alignments between SARS-CoV-2 and SARS-CoV showed the possibility of the insertions “NSPR” (Zhang C. et al., 2020) and “SPRR” (Xie et al., 2020) at the S1-S2 boundary. It has previously been reported that the sequence insertion at the S1-S2 boundary constitutes a furin cleavage site (Ord et al., 2020; Peacock et al., 2021). A comprehensive understanding of the consequence of the sequence insertion at the S1-S2 boundary is still missing, possibly because research focused on understanding the differences in the pathogenicity of the different SARS-CoV-2 variants and subvariants, which emerged rapidly. To determine whether the earlier SARS-CoV-2 isolate (USA/WA-CDC-WA1/2020 isolate, GenBank accession no. MN985325) has multiple novel sequence insertions in the S protein compared to SARS-CoV (Urbani strain, GenBank accession no. AY278741), we aligned the S protein sequences of both viruses using a constraint-based alignment tool for multiple protein sequences (COBALT) (Papadopoulos and Agarwala, 2007). We did not use MER-CoV for comparison because there is only 40% similarity between SARS-CoV-2 and MERS-CoV (Hu et al., 2020). Similar to a previous report (Zhang C. et al., 2020), we found sequence insertions (IS) in the SARS-CoV-2 S protein at four independent positions: IS1 “GTNGKTR,” IS2 “YYHK,” IS3 “HRSY,” and IS4 “NSPR” (Figures 1A, B). To determine whether any of these sequence insertions constituted or resembled any protein motifs, such as an NLS, we analyzed the SARS-CoV-2 S protein *in silico* with the PSORT II web portal for NLS prediction (Nakai and Horton, 1999). We found that the SARS-CoV-2 glycoprotein contained an NLS of the “pat7” motifs, one of the three NLS motifs described above (Supplementary Figure S1). To our surprise, the NLS motif “PRRARSV” was present at the proposed polybasic site and was due to the fourth sequence insertion “NSPR” (Figures 1A, B; Supplementary Figure S1). As expected, the NLS in the S protein was unique to SARS-CoV-2 among human pathogenic beta-coronaviruses, as neither the SARS-CoV S protein nor the MERS-CoV S protein has an NLS (Supplementary Figure S1).

### NLS-driven nuclear translocation of S protein (including S mRNA) occurs only in the SARS-CoV-2-infected airway epithelium

**Figure 2:**
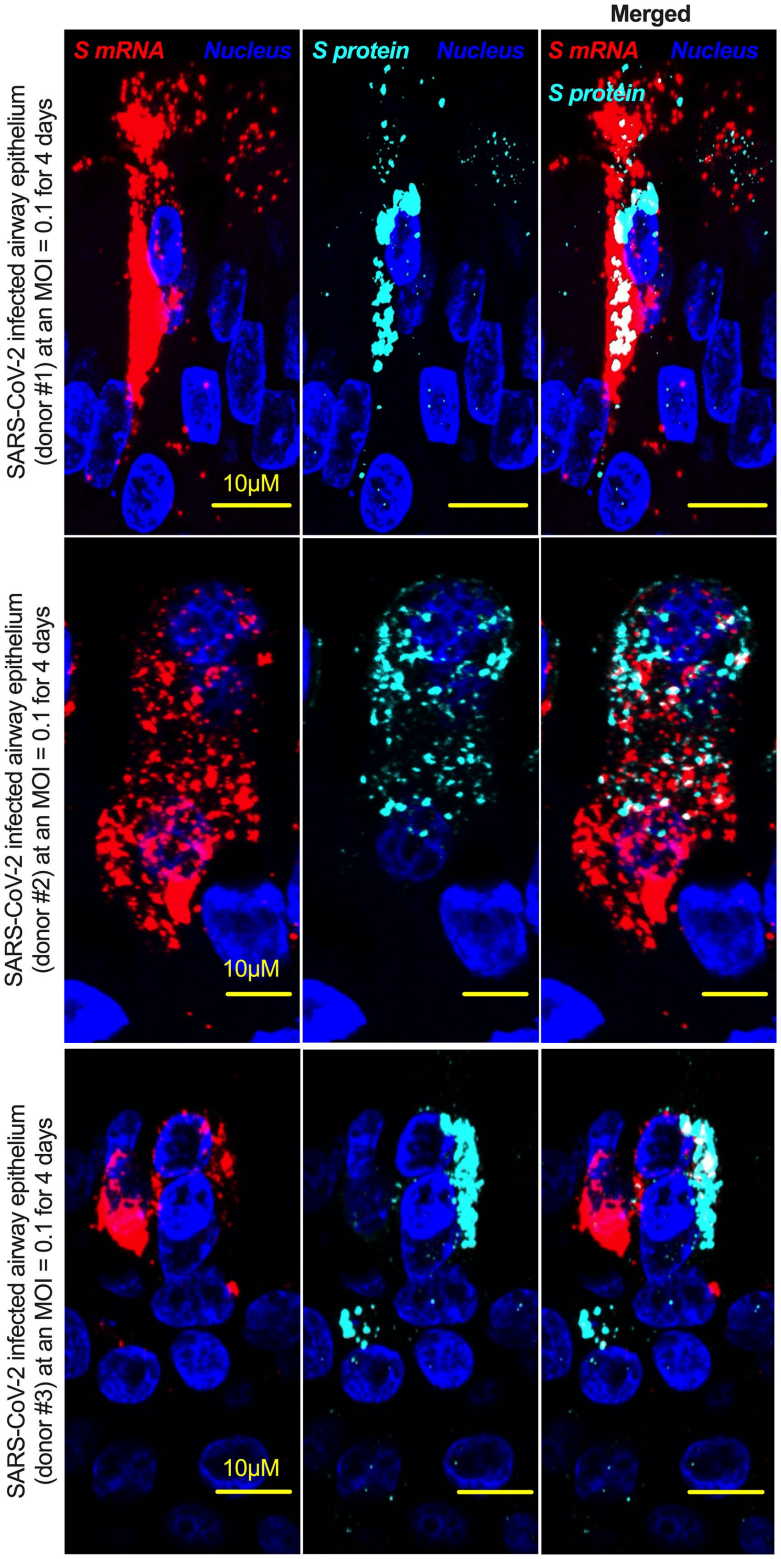
The intracellular distribution of S mRNA and S protein suggests nuclear translocation. Four-week highly differentiated pseudostratified airway epithelium was infected with SARS-CoV-2 at a MOI of 0.1 for 4 days, paraformaldehyde-fixed, paraffin-embedded, and sectioned at a thickness of 5 μm for immunohistochemistry (IHC) and slide preparation (Osan et al., 2020, 2021). A combined protocol of RNAscope and IHC was used to simultaneously detect S mRNA and S protein in the SARS-CoV-2-infected airway epithelium. S mRNA (red) was detected using a SARS-CoV-2 S mRNA probe for RNAscope, and S protein (cyan) was detected by an S protein-specific rabbit polyclonal antibody and a corresponding anti-rabbit secondary antibody for immunofluorescence (IFA) analysis. The nucleus (blue) was detected by DAPI staining. The images were taken under an Olympus confocal microscope using a 60x oil objective. The images represent multiple independent technical replicates from two independent experiments with different donors (experiment 1: donors 2 and 3 and experiment 2: donor 1). The scale bar is 10 μm.

**Figure 3:**
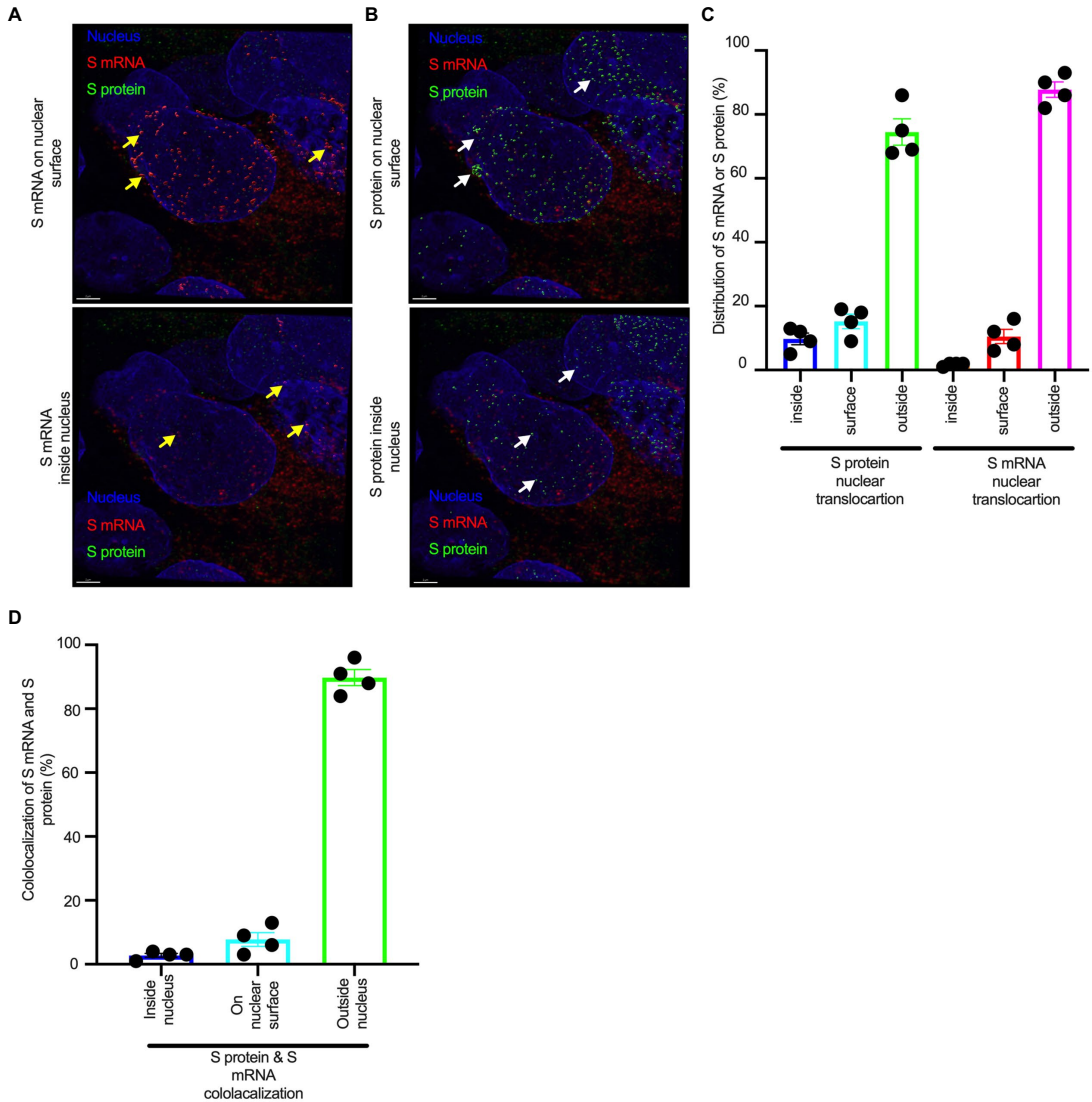
The nuclear translocation of S protein and S mRNA includes both the outer surface and inside of the nucleus. Separate slides (see Figure 2) were imaged under a Leica Stellaris confocal microscope (Leica) using a 63x oil objective. The images were then deconvolved using Huygen Essential deconvolution software (Scientific Volume Imaging). Using the surface rendering function of an image processing IMARIS software. **(A)** S mRNA (red) on the nuclear surface (top) and inside the nucleus (bottom). White arrows indicate S protein on the nuclear surface (top image) or inside the nucleus (bottom image). **(B)** S protein (green) on the nuclear surface (top image) and inside the nucleus (bottom image). White arrows indicate S protein on the nuclear surface (top image) or inside the nucleus (bottom image). **(C)** The total distribution of S mRNA and S protein in the cells. The data were obtained by combining multiple images from an independent experiment. **(D)** The total colocalization between S mRNA and S protein in the cells. The data were obtained by combining multiple images from an independent experiment.

Although viral glycoprotein nuclear translocation is rare, NLS-driven protein nuclear translocation has already been established in different viral infections (Ozawa et al., 2007; Boisvert et al., 2014). Thus, it is important to determine whether the SARS-CoV-2 S protein translocates into the nucleus in addition to its canonical cell surface localization through the ER-Golgi pathway. We hypothesized that the S protein could translocate into the nucleus in SARS-CoV-2-infected cells *via* the identified NLS motif (Rowland et al., 2005; Ozawa et al., 2007; Boisvert et al., 2014). We infected highly differentiated pseudostratified airway epithelial cells (which mimics *in vivo* human airway epithelium) with SARS-CoV-2 at a multiplicity of infection (MOI) of 0.1 for 4 days. First, we confirmed the presence of S mRNA and S protein in a 5 μm section of formalin-fixed paraffin-embedded SARS-CoV-2-infected cells by RNAscope and immunofluorescence analysis. Despite the rarity of viral mRNA (or even positive-strand RNA virus genome) to be nuclear (Whittaker and Helenius, 1998; Vargas et al., 2005), a recent study showed that SARS-CoV-2 mRNA accumulates in the nucleus of infected cells (Addetia et al., 2021). Our results showed that SARS-CoV-2 S mRNA was nuclear (Figure 2 left panel and merged images in the right panel). To confirm the physical apposition between S mRNA and the nucleus by comparing their distributions in fluorescent images, we used the spot-to-spot colocalization function in Imaris image analysis software (Oxford Instruments). We found that S mRNA was nuclear and abundant in the cytoplasm (Supplementary Figure S2, top panel, three donors). To avoid image artifacts, we imaged multiple independent slides of SARS-CoV-2-infected airway epithelium (from three independent donors) using at least two different high-end confocal microscopes. Additionally, we used at least two different image processing strategies to determine nuclear localization. Based on high-resolution imaging, we determined the subcellular distribution of S mRNA at near single-molecule and single-cell levels (Figures 2, 3A; Supplementary Figures S2, S3). Importantly, we were able to determine S mRNA nuclear translocation not only inside the nucleus but also on the nuclear surface (Figures 3A, C; Supplementary Figure S3). The determination of S mRNA distribution and abundance showed that S mRNA subcellular localization spans from the inside and outer surface of the nuclear membrane to everywhere in the cytoplasm. We found that almost 90% of S mRNA was distributed in the cytoplasm, which was expected, as SARS-CoV-2 transcription and replication occur in the cytoplasm (Figures 3A, C; Supplementary Figure S3). Interestingly, less than 10% of S mRNA was detected at the nuclear surface, which could explain the transitionary stage of S mRNA before it enters the nucleus or the novel transnuclear-membrane translocation of S mRNA, which was examined and described later (Figures 3A, C; Supplementary Figure S3). In approximately 1% of instances, S mRNA successfully translocated into the nucleus (Figures 3A, C; Supplementary Figure S3). The nuclear translocation of S mRNA is highly unusual because there have been few previous reports of S mRNA nuclear translocation and no information on the mechanism of nuclear translocation. However, we have explored how S mRNA could translocate into the nucleus.

We investigated whether the S protein translocated into the nucleus in the SARS-CoV-2-infected airway epithelium. Consistent with the S mRNA data, we found that the S protein translocated into the nucleus and was abundant on the cellular surface through the cytoplasmic ER-Golgi pathway (Figure 2, middle panel and merged images in the right panel). Based on high-resolution imaging, we determined that S protein nuclear translocation included both the inside of the nucleus and the nuclear surface (Figures 3B, C; Supplementary Figure S3). Similar to S mRNA quantification, we were able to quantify the distribution and abundance of S protein in the infected airway epithelium. We found that the S protein was distributed inside and in the outer membrane of the nucleus, the cytoplasmic ER-Golgi and the cell surface. We did not determine what portion of the S protein was localized inside and outside the cell surface. However, we quantified the subcellular distribution of the S protein inside the nucleus, outside the surface of the nucleus, and in the cytoplasm, which included cell surface expression because the S protein is a type 1 transmembrane glycoprotein. We found that approximately 75% of the S protein was distributed in sites other than the nucleus, including the cell surface and the cytoplasm, which was expected, as S protein translation and protein processing occur in the cytoplasm and *via* cytoplasmic ER-Golgi pathway, respectively (Figures 3B, C; Supplementary Figure S3). Interestingly, approximately 15% of the S protein was detected at the nuclear surface, which could explain the S protein transitionary stage before entering the nucleus or a novel transnuclear-membrane translocation of S protein, which was examined and described later (Figures 3A, C; Supplementary Figure S3). Interestingly, we found that a higher percentage of total S protein translocated into the nucleus than S mRNA (Figures 3A–C; Supplementary Figure S3). Although viral type-1 transmembrane glycoprotein translocation into the nucleus is rare, the NLS in the S protein is responsible for nuclear translocation. It was apparent that NLS-driven S protein nuclear translocation was SARS-CoV-2 specific, and a side-by-side infection experiment with both viruses showed that the S protein of SARS-CoV did not translocate into the nucleus (Supplementary Figure S4). As both S mRNA and S protein translocated into the nucleus, it is important to determine whether S mRNA and S protein colocalize in different subcellular sites.

### Colocalization of S mRNA and S protein in different subcellular locations in the SARS-CoV-2-infected airway epithelium

**Figure 4:**
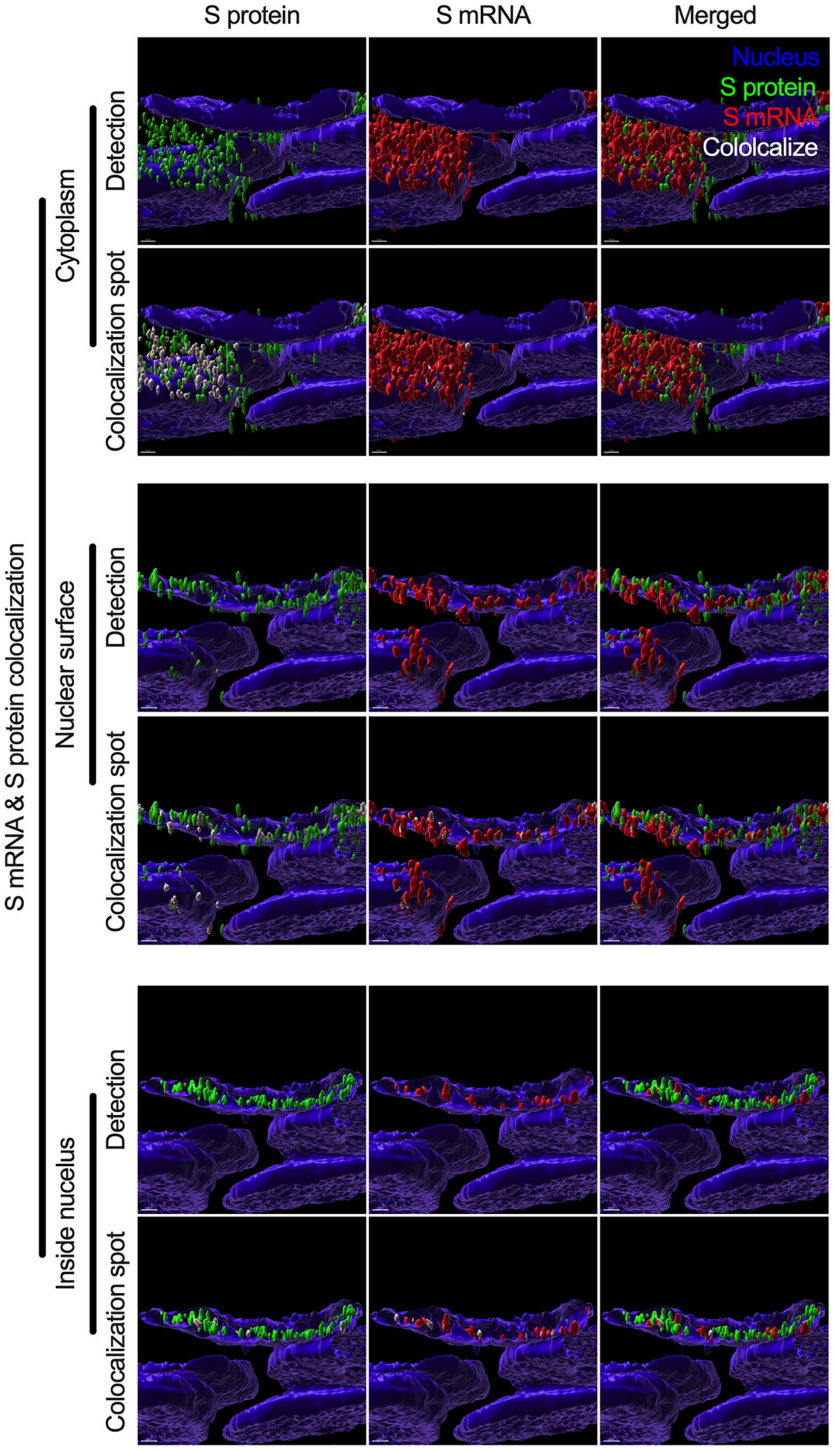
Colocalization between S mRNA and S protein inside infected cells. The images (see Figure 3) were analyzed by using the surface rendering and colocalization features of IMARIS. S protein and S mRNA distribution and colocalization in the cytoplasm (top panel), on the nuclear surface (middle panel) and inside the nucleus (bottom panel). The specific region of colocalization is indicated by a white spot. Scale bar 0.5 μm.

While we can explain S protein nuclear translocation due to the presence of an NLS motif in the amino acid sequence, we can only hypothesize that S mRNA nuclear translocation is possible due to a direct interaction between S protein and S mRNA, which can be explained by the colocalization between them. The SARS-CoV-2 N protein is an abundant RNA binding protein that is essential for viral genome packaging (Cubuk et al., 2021). While the structural basis of N protein binding to single-or double-stranded RNA is known (Dinesh et al., 2020), there is no information about whether S protein binds to S mRNA. As we found similar intracellular distribution of both S mRNA and S protein, we hypothesized that S protein interacts with S mRNA to translocate the protein–mRNA complex to different subcellular locations, including the cytoplasm and nucleus, but not the cell surface. By examining the colocalization between the S protein and S mRNA, we could confirm the presence of the protein–mRNA complex in the SARS-CoV-2-infected airway epithelium. Here, we refer to colocalization as an association between S mRNA and S protein at different intracellular locations. Technically, two separate fluorescence molecules that emit different wavelengths of light are superimposed within an indeterminate microscope resolution. To determine whether S mRNA and S protein colocalize, we used a high-resolution imaging strategy. We quantified the colocalization on a percentage scale. We found that approximately 85% of the colocalization, which was the highest, was observed outside the nucleus (Figure 3D). These data are consistent with the previously described spatial expression data of both S protein and S mRNA (Figures 3A–C). As expected, lower and the lowest percentages of colocalization between the S protein and S mRNA were observed on the nuclear surface and inside the nucleus, respectively (Figure 3D). We were able to pinpoint the colocalization site by using high magnification confocal imaging followed by image processing. Representatives of S mRNA and S protein colocalization in the cytoplasm (Figure 4, top two panels), on the surface of the nuclear membrane (Figure 4, middle two panels), or inside the nucleus (Figure 4, bottom two panels) are shown. We observed S protein and S mRNA colocalization in three subcellular locations, which was confirmed at the single-cell level in SARS-CoV-2-infected cells (Supplementary Figure S5). We also observed that S mRNA inside and on the nuclear surface was associated with the S protein, in contrast to the cytoplasmic S mRNA distribution with or without colocalization with the S protein (Figure 4; Supplementary Figure S5). Thus, S mRNA translocates into the nucleus through the S protein-S mRNA complex and is driven by the S protein (Figure 4; Supplementary Figure S5).

### SARS-CoV-2 S protein’s transient expression confirms its nuclear translocation in the transfected cells

**Figure 5:**
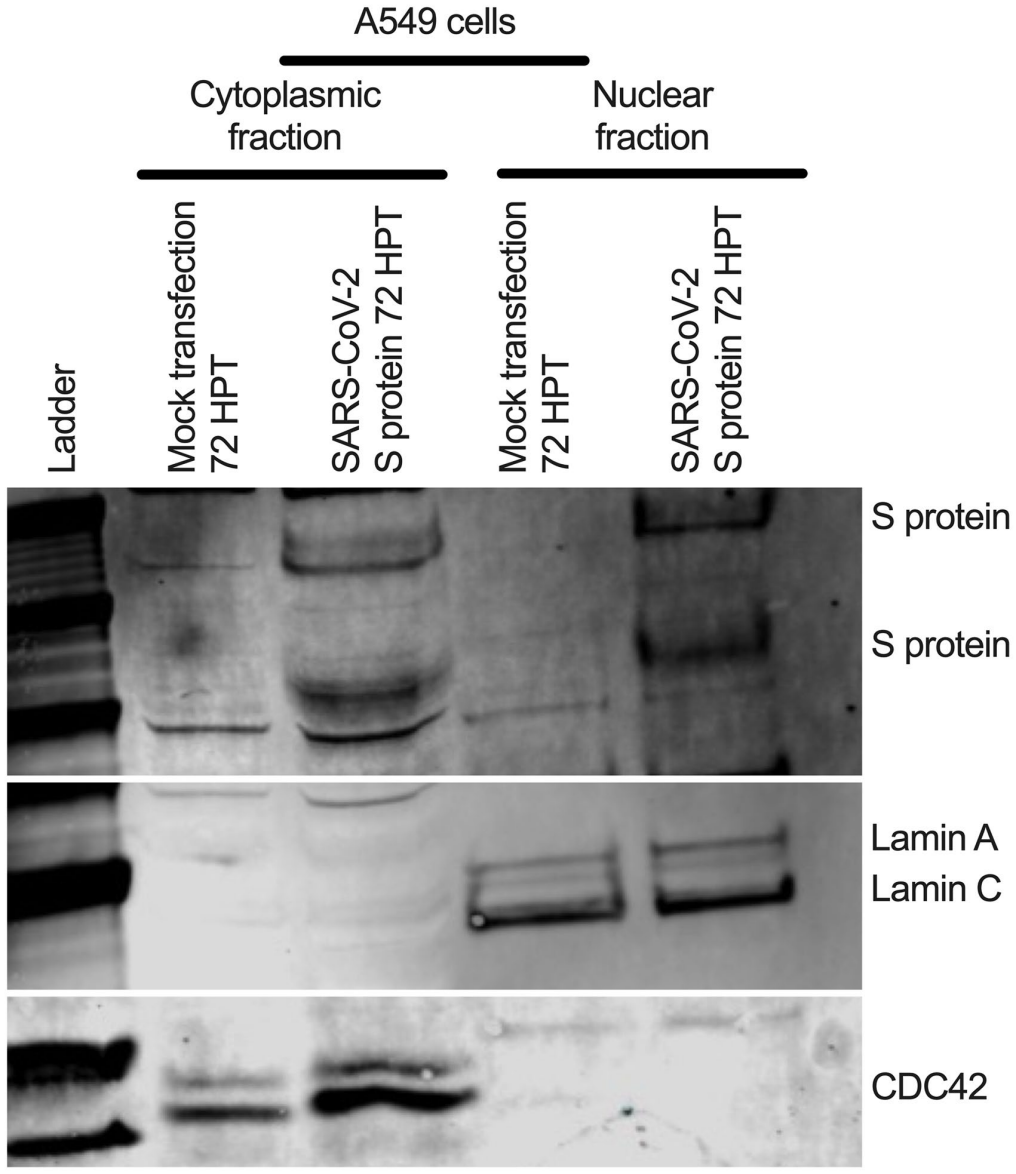
S protein in both cytoplasmic and nuclear fraction of the transfected A549 cells. A549 cells were transfected with SARS-CoV-2 S plasmid (Leventhal et al., 2021) for 72 h. SARS-CoV-2 S protein was detected in both cytoplasmic and nuclear fractions by Western blotting. Cdc-42 detection confirms cytoplasmic portion and Lamin A and C detection confirms nuclear fraction.

To determine whether SARS-CoV-2 S protein’s nuclear translocation is independent of other viral protein’s involvement, we transfected primary normal human bronchial epithelial (NHBE) cells with a recombinant plasmid containing codon-optimized full-length DNA sequence of SARS-CoV-2 S protein (Leventhal et al., 2021) for 48 h. We used an immunofluorescent assay (IFA) to detect the S protein in the transfected cells. We found a broader subcellular distribution of S protein (Supplementary Figure S6A). Both vertical and horizontal cross-sections of the epifluorescence images showed that the fluorescence signal of the S protein overlapped the nuclear stain. However, it was prudent to determine whether the observed S protein nuclear distribution is independent of its abundant endoplasmic reticulum (ER) distribution, as it is processed there before translocating to the cell surface. Thus, we further investigated the S protein’s nuclear translocation in the lung epithelial A549 cells. We first co-stained the S protein and a common ER protein, calnexin. We found that S protein sub-cellular distribution beyond ER, which complemented our previous finding of its nuclear translocation (Supplementary Figure S6B). However, we need to show S protein translocated into the nucleus in the transfected A549 cells. We co-stained the S protein and lamin B1, a nuclear inner membrane protein. We found that S protein fluorescence overlapped with the lamin B1 suggesting S protein’s nuclear translocation in the transfected A549 cells (Supplementary Figure S6C). S protein nuclear distribution was distinct from the eGFP’s diffusive expression throughout the cells (Supplementary Figure S6C). In addition to a fluorescent-based detection strategy, we used a biochemical approach to confirm the S protein’s nuclear translocation. We hypothesized that the SARS-CoV-2 S protein should be detected in nuclear and cytoplasmic fractionating to the SARS-CoV-2 S plasmid transfected cells. We transfected A549 cells with the same plasmid for 72 h. For control, we similarly mock-transfected A549 cells without the plasmid. We collected cytoplasmic and nuclear fractions of the S protein plasmid transfected or mock-transfected using ReadiPrep Nuclear/Cytoplasmic Fraction kit (AAT Bioquest) according to the manufacturer’s instructions. We found S protein in both the nuclear and cytoplasmic fractions of the transfected A549 cells (Figure 5). We detected lamin A and C and cell division cycle protein 42 (Cdc42) on the same gel to confirm no mix-up between nuclear and cytoplasmic portions. While lamin A and C were detected in the nuclear portion, Cdc42 was detected only in the cytoplasmic portion (Figure 5). Our results suggested that transient expression of the SARS-CoV-2 S protein caused its wider subcellular distribution, including the nucleus in the transfected cells.

### NLS-driven N protein nuclear translocation is common in SARS-CoV, MERS-CoV, and SARS-CoV-2 infections

**Figure 6:**
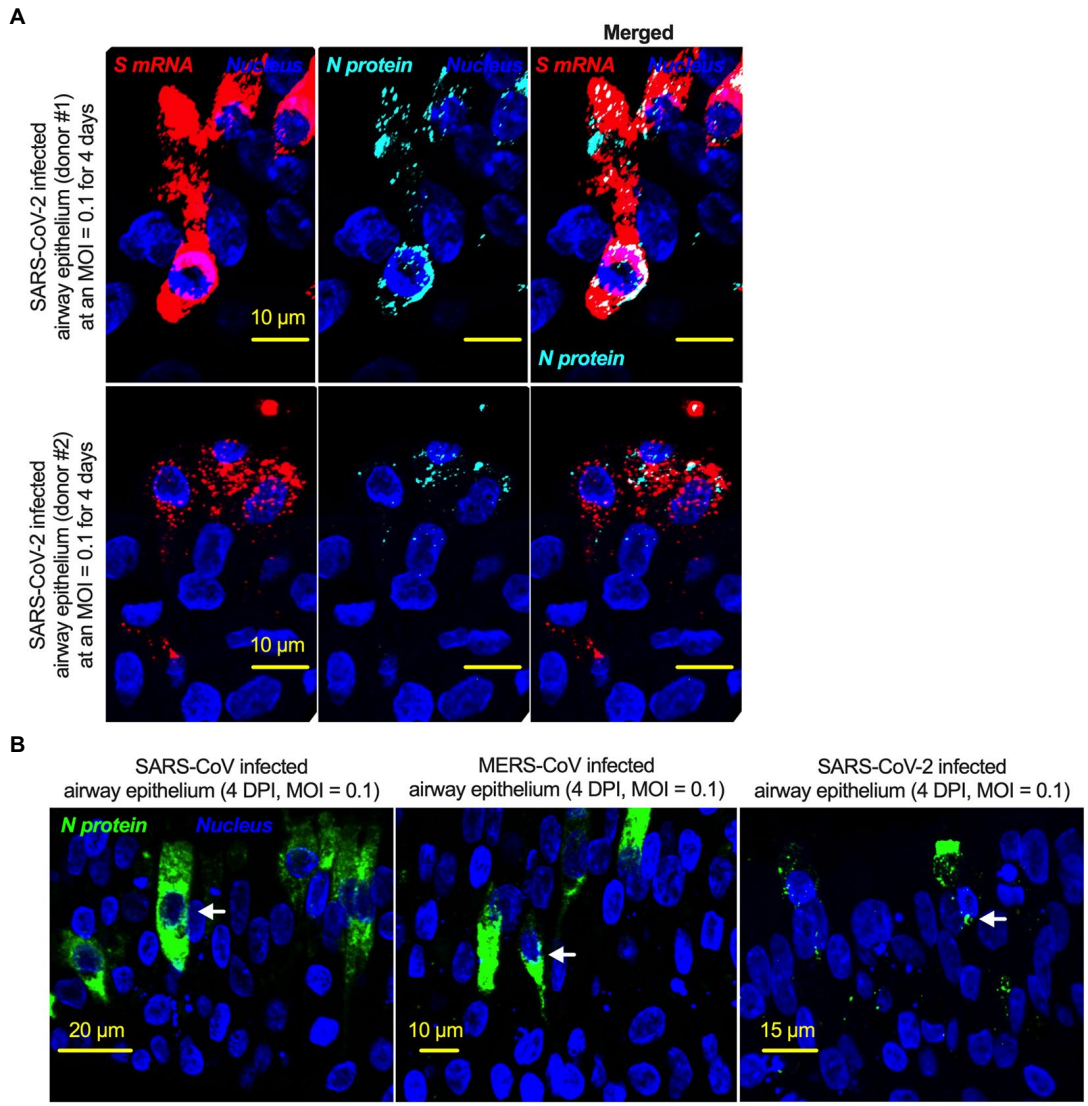
The nucleoproteins of SARS-CoV, MERS-CoV, and SARS-CoV-2 translocate into the nucleus. **(A)** Four-week pseudostratified airway epithelium was infected with SARS-CoV-2 at a MOI of 0.1 for 4 days, fixed, paraffin-embedded, and sectioned at a thickness of 5 μm for immunohistochemistry slide preparation. Simultaneous detection of S mRNA (shown in red) and N protein (shown in cyan) on the same slide was performed by a combined detection protocol in RNAscope-based mRNA and immunofluorescence-based protein detection. An S mRNA-specific probe was used for RNAscope, and an N protein-specific rabbit polyclonal antibody and the corresponding anti-rabbit secondary antibody were used. The nucleus (shown in blue) was detected by DAPI staining. The images were taken under an Olympus confocal microscope using a 60x oil objective. The images represent multiple independent technical replicates from two independent, healthy donors (top row: donor #1 and bottom row: donor #2). The scale bar is 10 μm. **(B)** Four-week pseudostratified airway epithelium was infected with SARS-CoV-2, SARS-CoV, or MERS-CoV at an MOI of 0.1 for 4 days. SARS-CoV-2 or SARS-CoV N protein was detected by an N protein-specific rabbit polyclonal antibody and corresponding anti-rabbit secondary antibody. Similarly, the MERS N protein was detected by MERS N protein-specific primary and corresponding secondary antibodies. The nucleus (shown in blue) was detected by DAPI staining. The images represent multiple independent technical replicates from one experiment (donor #1).

There is no comprehensive information on SARS-CoV-2 N protein NLS motifs, and we already know that other pathogenic coronaviruses, particularly SARS-CoV (Rowland et al., 2005; Timani et al., 2005) and MERS-CoV (Yang et al., 2013) N proteins, have NLSs. Therefore, we searched for NLSs in the SARS-CoV-2 N protein by using the PSORT II. We found that the SARS-CoV-2 N protein has 7 NLSs covering all three types of NLS motifs (pat4: 2; pat7: 3, and bipartite: 2); however, the SARS-CoV N protein has 8 NLS motifs (pat4: 2; pat7: 4, and bipartite: 2) (Supplementary Figure S7). N protein translocation into the nucleus or at least the perinuclear region was confirmed (Figure 6A; Supplementary Figure S8). However, SARS-CoV-2 N protein nuclear translocation was not as robust as SARS-CoV nuclear translocation (Figure 6B; Supplementary Figure S8). The reduced nuclear translocation of the SARS-CoV-2 N protein is probably due to the absence of one pat7 NLS motif in the SARS-CoV-2 N protein compared to that in SARS-CoV (Supplementary Figures S8, S9).

## Discussion

In the context of SARS-CoV, one of the controversies regarding the natural origin of SARS-CoV-2 is that its S gene has multiple novel sequence insertions. Zhang C. et al. analyzed the report by Pradhan et al. (withdrawn) (Pradhan et al., 2020) on the presence of four unique novel sequences in the SARS-CoV-2 S gene and showed that these four sequence insertions were not related to the receptor-binding domain (RBD) (Zhang C. et al., 2020). A recent study identified S gene novel sequence insertions among several key genomic features that differentiate SARS-CoV-2 from other beta-coronaviruses, particularly SARS-CoV and MERS-CoV (Gussow et al., 2020). The source and characterization of these sequence insertions have yet to be determined; however, the closest BLAST hit of these sequences is bat coronavirus RaTG13 (Gibson et al., 1992). Similar to a previous report (Zhang C. et al., 2020), we found four multiple sequence insertions in the SARS-CoV-2 S protein: IS1 “GTNGKTR,” IS2 “YYHK,” “HRSY,” and IS4 “NSPR” (Figure 1). Here, we showed that the fourth novel sequence insertion in the S gene created a functional pat7 NLS motif and resulted in nuclear translocation of the S protein, which not only complemented previous *in silico* findings (Gussow et al., 2020) but also identified a novel pathogenic genomic feature of the S gene. Surprisingly, the fourth sequence insertion has received attention due to the description of a proposed polybasic furin cleavage site “RRAR,” which may contribute to increased serin protease-driven entry of SARS-CoV-2 (Andersen et al., 2020) and is implicated in broader tropism and/or enhanced viral transmissibility compared to SARS-CoV (Walls et al., 2020). Interestingly, the proposed SARS-CoV-2 furin cleavage site (P-R-R-A-R) considered as noncanonical, as a consensus recognition sequences for furin proteases is (X-Arg-X-Lys/Arg-Arg-X) (Klimstra et al., 1999; Chan and Zhan, 2022). Thus a generic furin consensus cleavage site motif should contain the canonical four amino acid motif R-X-[K/R]-R, although R-X-X-R is the minimal cleavage site on the substrate for successful furin cleavage (Krysan et al., 1999; Tian et al., 2011). However, we found that the IS4 “NSPR” created a pat7 NLS “PRRARSV” in the S protein, which was unique to SARS-CoV-2. Whether this noncanonical furin cleavage destroys the function of the NLS motif is important to determine, as the proposed noncanonical furin cleavage site is constitutively within the NLS motif (described later). Due to the specificity of the amino acid motif, a furin cleavage motif is not expected to fulfill the characteristics of an NLS motif. We first reported that the S protein translocated into the nucleus in the SARS-CoV-2-infected airway epithelium, which is an appropriate lung model for studying respiratory virus infection *in vitro* (Osan et al., 2021, 2022). Our results confirmed that the SARS-CoV-2 S protein was a unique addition to the list of viral proteins that possess NLSs and consequently translocate into the nucleus of infected cells (Rowland et al., 2005; Ozawa et al., 2007; Parent, 2011; Boisvert et al., 2014). We also confirmed S protein’s nuclear translocation primarily due to its NLS motif, as transient expression of S protein in either primary NHBE cells or A549 cell line showed its broader subcellular distribution including nucleus. Among coronaviruses, SARS-CoV-2 S protein is the first type-1 transmembrane glycoprotein that translocates into the nucleus. Vesicular stomatitis virus (VSV), which is a negative sense RNA virus, has a glycoprotein that translocates to the nucleus as well. A study by the University of Illinois at Urbana Champaign showed exactly how the glycoprotein on VSV was able to travel to the nucleus of hamster kidney cells (DaPoian et al., 1996).

NLS-driven S protein nuclear translocation is a novel feature of SARS-CoV-2 infection compared to other pathogenic coronaviruses. However, the pathogenic contribution of the S protein’s NLS motif to virus-induced pathophysiology is yet to be determined. Our results suggested that the S protein translocated into the nucleus due to the NLS, which also raised two important points. First, we investigated whether the proposed polybasic site “RRAR” could itself be an NLS motif. The answer was that the proposed polybasic site was not an NLS motif because an NLS is a well characterized and predefined amino acid sequence motif (Robbins et al., 1991; Hicks and Raikhel, 1995; Rowland et al., 2005). Additionally, the amino acid sequence of the probable sequence insertion “NSPR” (Zhang C. et al., 2020) was also not an NLS but was part of the P7 “PRRARSV” NLS. Thus, the inserted sequence creates the NLS in the S protein of SARS-CoV-2 and may make SARS-CoV-2 unique among human pathogenic coronaviruses.

The second important point was whether the NLS motif was functional in the context of the described polybasic site at the S1/S2 boundary. All type-1 transmembrane glycoproteins are processed through the ER-Golgi pathway before signal peptide-driven cellular surface localization. The proposed polybasic site was functional (availability to proteases) when the S protein was on the virion for host cell entry. A fully posttranslationally processed S protein surface translocation could also provide a polybasic site to be processed by furin cleavage. However, there is no information on the availability or usability of the S protein’s polybasic site by furin proteases in the cytoplasm before virus assembly. Thus, the NLS is functional in SARS-CoV-2-infected cells, and the polybasic site only functions during the viral entry step. The NLS is obviously functional in infected cells, and no furin cleavage at the polybasic site is necessary other than for viral entry. Our results confirmed that the S protein NLS motif was functional in SARS-CoV-2-infected cells. Although mutating the polybasic site (which also mutated the NLS) may impact viral S protein function *in vitro*, the result will not confirm or deny that one is more important than the other between the polybasic site and the NLS. While our result does provide direct evidence for the presence of the NLS motif and nuclear translocation of the S protein, our results do not confirm nor deny that the NSPR sequence has a natural origin. Instead, our results showed that the inserted sequence NSPR created a functional NLS motif, which increased the intracellular distribution of the S protein, including novel nuclear translocation. The novel nuclear translocation of the SARS-CoV-2 S protein suggests that: 1. the nuclear translocation of the S protein reduces its surface expression, but whether it contributes to evading host immune recognition remains to be determined; and 2. the colocalization of the S protein with S mRNA suggests that the S protein has an RNA binding motif, which remains to be determined. One of the important ways of confirming a functional NLS motif is to use site-directed mutational analysis. Plasmid-driven transient expression of S protein in the human lung airway A549 cell line and primary normal human branchial epithelial cells showed robust S expression but was toxic to the cells. Therefore, the success of site-directed mutational analysis of the S protein in a transient expression system is doubtful and the characterization of NLS by a mutational analysis is yet to be determined. Thus, our novel findings emphasize further research on the NLS motif of the SARS-CoV-2 S protein.

One of the most important findings in our study was the simultaneous detection of the different spatial distributions of S protein and S mRNA at near single-molecule level in a single infected cell. We confirmed that S mRNA translocated into the nucleus by image analysis of the colocalization of S mRNA with nuclear staining. When SARS-CoV-2 N protein has already been shown to bind to RNA (Schmidt et al., 2021), we still do not know how exactly S protein binds to S mRNA for nuclear translocation. We only know is S mRNA nuclear translocation is more likely mediated by the S protein because S mRNA nuclear translocation was always associated with the S protein. Importantly, S mRNA colocalized with the S protein in the cytoplasm and nucleus (both inside and outside the surface of the nucleus). Our results support a previous machine learning based study findings SARS-CoV-2 RNA genome and sub-genomic RNAs can be translocated in the host cells’ mitochondrial matrix and nucleus (Wu et al., 2020). Our results suggest that around 1% S mRNA translocated into the nucleus. S mRNA’s (potentially SARS-CoV-2 mRNA genome) subcellular localization may play a significant role in SARS-CoV-2 pathogenesis. To determine whether the SARS-CoV-2 genome interacts with either S protein or N protein, we *in silico* analyzed RNA-protein interactions using the RPISeq web portal, which offers the only sequence-based prediction model (Muppirala et al., 2011). We found that both S and N protein binding probability to the SARS-CoV-2 genome scored exactly 1 (Dataset S1 and S2). SARS-CoV-2 N protein is an abundant RNA binding protein essential for viral genome packaging (Cubuk et al., 2021). While the structural basis of N binding to single or double-stranded RNA is known (Dinesh et al., 2020), we found that S mRNA nuclear translocation aids *via* S protein. However, the mechanism of S protein binding to the mRNA or possibly positive-strand RNA genome is yet to be determined. Although the primer-probe was designed to target S mRNA, the SARS-CoV-2 positive-strand RNA genome (whole or partial) can be targeted by the same probe due to the sequence similarity between S mRNA and the whole or partial genome. Thus, our results lack sufficient detail contributing to the discussion of the controversial scientific topic of whether there is any possibility of SARS-CoV-2 genome integration into the host DNA (Smits et al., 2021; Zhang et al., 2021). Additionally, one of the significant differences in the S protein sequences of SARS-CoV and SARS-CoV-2 is the pat7 NLS motif. Our results showed that transient expression of S protein by a plasmid containing the full-length S protein DNA sequence in both primary NHBE cells and A549 cell line lead its wider subcellular distribution including nucleus. Whether S protein expression by the current vaccine platforms causes suboptimal expression of S protein on the cell surface due to the NLS remains to be determined.

In conclusion, the SARS-CoV-2 S protein has a functional pat7 NLS “PRRARSV,” that results in one out of four S proteins translocating into the nucleus in infected cells. S Protein appears to shuttle S mRNA (possibly the genome) into the nucleus of the infected cells. Transient expression of full-length S protein in A549 cells reveals its nuclear translocation suggesting S protein’s nuclear translocation is NLS-driven and independent of interacting with another viral protein. Thus NLS of the S protein may contribute to the evasion of the host immune response and is a novel feature of SARS-CoV-2.

## Materials and methods

### Cells and viruses

Primary normal human bronchial epithelial (NHBE) cells from healthy adults and high-risk adults (deidentified) were obtained from Dr. Kristina Bailey at the University of Nebraska Medical Center (UNMC) (Omaha, NE) under an approved material transfer agreement (MTA) between the University of North Dakota (UND) and UNMC (Omaha, NE). The protocol for obtaining cells was reviewed by the UNMC IRB and was determined to not constitute human subject research (#318-09-NH). In this study, we used cells from three donors: nonsmoker healthy adults (donors #1 and #2) and adult with chronic obstructive pulmonary disease (COPD) (donor #3). The protocols for subculturing primary NHBE cells were published previously (Osan et al., 2020, 2021, 2022; Talukdar et al., 2022). Human lung epithelial A549 cells was maintained in F-12 medium (Thermo Fisher Scientific) as described previously (Mehedi et al., 2016). SARS-CoV-2 (USA/WA-CDC-WA1/2020 isolate, GenBank accession no. MN985325; kindly provided by CDC), SARS-CoV (Urbani strain, GenBank accession no. AY278741; kindly provided by Rocky Mountain Laboratories (RML), NIAID, NIH), and MERS-CoV (GenBank accession no. NC_019843.3; kindly provided by the Department of Viroscience, Erasmus Medical Center, Rotterdam, The Netherlands) were used for *in vitro* infections described below.

### *In silico* analysis

We have used open-source web portals for different *in silico* analyses. 1. Constraint-based alignment tool for multiple protein sequences (COBALT)^1^ was used for multiple sequence alignment. 2. PSORT II^2^ was used for NLS prediction. 3. The RPIseq web portal^3^ was used for RNA–protein interactions.

### Highly differentiated pseudostratified bronchial airway epithelium

The protocols for differentiating primary NHBE cells to form a pseudostratified bronchial airway epithelium were published previously (Osan et al., 2020, 2021, 2022; Talukdar et al., 2022). Briefly, Transwells (6.5 mm) with 0.4-μm-pore polyester membrane inserts (Corning Inc.) were coated with PureCol (Advanced BioMatrix) for 20 min before cell seeding. NHBE cells (5×10^4) suspended in 100 μL of complete airway epithelial cell (cAEC) medium [AEC medium (Promocell) + SupplementMix (Promocell) + 1% penicillin–streptomycin (V/V) (Thermo Fisher Scientific) + 0.5% amphotericin B (V/V) (Thermo Fisher Scientific)] were seeded in the upper chamber of the Transwell. Then, 500 μL of cAEC medium was added to the lower chamber of the Transwell. When the cells formed a confluent layer on the Transwell insert, the cAEC medium was removed from the upper chamber, and in the lower chamber, the cAEC medium was replaced with complete ALI medium [PneumaCult-ALI basal medium (Stemcell Technologies Inc.) + with the required supplements (Stemcell Technologies) + 2% penicillin–streptomycin (V/V) + 1% amphotericin B (V/V)]. The complete ALI medium in the lower chamber was changed every day. The upper chamber was washed with 1x Dulbecco’s phosphate-buffered saline (DPBS) (Thermo Fisher Scientific) once per week initially but more frequently when more mucus was observed during later days. All cells were differentiated for at least 4 weeks at 37°C in a 5% CO_2_ incubator. We observed motile cilia in the differentiated airway epithelium similar to previously described (Osan et al., 2020).

### Viral infection

All viral infection experiments were conducted in the high biocontainment facility at RML, NIAID, NIH, Hamilton, MT. After approximately 3 weeks, the differentiated airway epithelium on Transwells was shipped to RML in an optimized transportation medium (Osan et al., 2020, 2022), and the recovered cells were maintained in complete ALI medium for approximately 1 week before infection. For infection, the airway epithelium on Transwells was washed with 200 μL of 1x PBS to remove mucus and were infected on the apical site with SARS-CoV-2, MERS-CoV, or SARS-CoV at a MOI of 0.1 in 100 μL 1x PBS for 1 h (at 37°C with 5% CO_2_). For mock infection, the Transwells were similarly incubated with 100 μL 1x PBS without virus. The viral inoculum was then removed, and the epithelium on the Transwell was washed twice with 200 μL of 1x PBS. Complete ALI medium (1,000 μL) was added to the lower chamber of each Transwell, and the upper chamber was kept empty. Mock-infected and virus-infected Transwells were incubated for 4 days at 37°C in an incubator with 5% CO_2_ (Osan et al., 2022).

### Paraformaldehyde (PFA) fixation and paraffin embedding

At 4 days postinfection (DPI), 200 μL of 1x PBS was added to the apical site of the Transwell for washing before PFA fixation. In the basal side of the Transwell inserts was 200 μL of 1x PBS. For PFA fixation, 200 μL of 4% PFA (Polysciences) was added to the upper chamber of the Transwells and incubated for 30 min, and the Transwells were further maintained overnight in 4% PFA prior to removal from high biocontainment. The PFA fixation protocol was approved as an inactivation method for coronaviruses by the RML Institutional Biosafety Committee. The PFA-fixed airway epithelium was paraffin-embedded and sectioned at a thickness of 5 μm for slide preparation as previously described (Osan et al., 2021).

### Simultaneous detection of S mRNA and S protein

Slides with 5 μm sections were first deparaffinized by incubation in a Coplin jar as follows: 1. Histo-Clear for 5 min, two times; 2. 100% ethanol for 5 min, three times; 3. 95% ethanol for 5 min, 4. 70% ethanol for 5 min, and 5. distilled water for 5 min. The deparaffinized slides were immediately incubated in 0.5% Triton X-100 in 1x PBS for 30 min. The slides were washed three times with 1X PBST (1x PBS with Tween 20) or 1x PBS for 5 min. A hydrophobic barrier was drawn around the 5 μm section on the slides by using an Immedge Hydrophobic Barrier Pen. To reduce nonspecific antibody binding, the section was blocked with 10% goat serum (Vector Laboratories) in 1x PBST for 2 h at 4°C. The slides were then incubated with viral protein-specific primary antibody solution in 1x PBST (e.g., SARS-CoV/SARS-CoV-2 S protein-specific rabbit polyclonal antibody at a 1:100 dilution, SARS-CoV/SARS-CoV-2 N protein-specific mouse monoclonal antibody at a 1:100 dilution, or MERS-CoV N protein-specific mouse monoclonal antibody at a 1:100 dilution) (Osan et al., 2021) overnight at 4°C. The slides were then incubated with the corresponding secondary antibody solution (anti-mouse or anti-rabbit AF488 or AF647, Thermo Fisher Scientific) in 1x PBST for 2 h at room temperature. We then stained the nuclei with DAPI reagent (Advanced Cell Diagnostics) or used RNAscope multiplex V2 to detect SARS-CoV-2 S mRNA (Probe V-nCoV2019-S) according to the manufacturer’s instructions (Advanced Cell Diagnostics). The sections were mounted on Tech-Med microscope slides (Thomas Fisher Scientific) using ProLong-Gold antifade mounting medium (Thermo Fisher Scientific).

### Imaging and image analysis

For the immunofluorescence (IFA)-based detection of SARS-CoV-2/ SARS-CoV S and N protein were detected by using S-specific rabbit polyclonal antibody (Thermo Fisher Scientific) and N-specific mouse monoclonal antibody (Thermo Fisher Scientific), respectively. Similarly, MERS-CoV N protein was detected using N-specific mouse monoclonal antibody (Sino Biological). Similarly, calnexin and lamin B1 were detected using calnexin-specific mouse monoclonal antibody (Antibody) and lamin B1 specific mouse monoclonal antibody (Abcam), respectively. The slides were then incubated with the corresponding secondary antibody solution (anti-mouse or anti-rabbit AF488 or AF647, Thermo Fisher Scientific). For primary antibody we used 1:1000 dilution, whereas we used 1:200 dilution for secondary anybody incubation. Rhodamine Phalloidin (Cytoskeleton Inc.) (1:500 dilution) and DAPI (Thermo Fisher Scientific) (1 drop in 1,000 μL) were used for staining F-actin and nucleus, respectively. For mounting coverslip on a cover glass, we used ProLong Gold Antifade Msounting medium (Thermo Fisher Scientific). The images were taken under an Olympus FluoView laser scanning confocal microscope (Olympus FV3000) enabled with a 60X objective (Olympus), a Leica Stellaris confocal microscope (Leica Microsystem) using a 63x oil objective or a Leica DMI8 epifluorescence microscope (Leica Microsystem). The images were then deconvolved using Huygen Essential deconvolution software (Scientific Volume Imaging). The surface rendering function of Imaris image processing software (Oxford Instruments) was used. The images were also analyzed for spot-to-spot colocalization by Imaris. Where applicable, images taken under a Leica DMI8 microscope were processed using 3D deconvolution and 3D view modules in LASX software (Leica Microsystem). For figure preparation, Prism version 9 (GraphPad) and Adobe Photoshop (Creative Cloud) software were used.

### Transient transfection

NHBE cells (two independent donors #1 and #2) were seeded on 24 well plate in cAEC medium at 37°C in a 5% CO_2_ incubator. Cells were then transfected with SARS-COV-2 Spike (S) expressing plasmid (generous gift from Shanna S Leventhal and David M Hawman) (Leventhal et al., 2021) (0.5 μg) using TransfeX transfection reagent (ATCC) according to the manufacturer instructions. Similarly, A549 cells were seeded on a 24 well plate in F-12 medium at 37°C in a 5% CO_2_ incubator. For imaging purpose, a coverslip was previously added onto the well of 24-well plate before seeding cells. The cells were transfected with SARS-COV-2 Spike (S) expressing plasmid (0.5 μg) using TransLT-1 transfection reagent (Mirus Bio LLC) according to the manufacturer instructions. For control, eGFP in pCAGGS plasmid (generous gift from Dr. Thomas Hoenen) was transfected similarly. For mock-transfection, cells were treated similarly without any plasmid.

### Nuclear and cytoplasmic fractionation and Western blotting

Old medium was removed, and cells were washed at 1x PBS. Both cytoplasmic and nuclear portion from the S-transfected or mock-transfected A549 cells were collected using nuclear fractionating kit according to the manufacturer instructions. Around 30 ul of cytoplasmic and nuclear portions were run 4–12% bis-tris gel (Thermo Fisher Scientific) and followed by transferred on polyvinylidene fluoride (PVDF) membrane (Thermo Fisher Scientific) using iblot (Thermo Fisher Scientific). The membrane was blocked using Li-Cor blocking buffer (LI-COR Biosciences). For SARS-CoV-2 S protein was detected using SARS-CoV/SARS-CoV-2 S protein-specific rabbit polyclonal antibody (Thermo Fisher Scientific) at a 1:2500 dilution. Lamin A and C were detected protein-specific rabbit polyclonal antibody (Abcam) (molecular weight around 75 kDa and lower, respectively) at a 1:5000 dilution. Cdc42 were detected protein-specific rabbit polyclonal antibody (Cell Signaling Inc) (molecular weight around 20 kDa) at a 1:1000 dilution. A sequential different primary and secondary antibodies (e.g., anti-rabbit goat IRDye 680RD or ant-rabbit goat IRDye 800CW incubation for different protein detection on the same gel. For accuracy of protein detection, individual protein specific gel was also generated.

## Data availability statement

The original contributions presented in the study are included in the article/Supplementary material, further inquiries can be directed to the corresponding author.

## Author contributions

MM conceived the project and designed all the experiments, analyzed (*in silico*) the viral genome and protein sequences, and prepared the figures and wrote and edited the manuscript. KB provided the primary cells. FF performed the viral infection work. KJ, SS, and MM performed all staining for detection. MM and SS generated the microscopic images. JK and MM processed and quantified images. All authors contributed to the article and approved the submitted version.

## Funding

This work was funded by the NIH/NIGMS awards P20GM113123 and T34GM122835, VA grant 101-BX005413 and partially by the Intramural Research Program, NIAID, NIH. The content is solely the responsibility of the authors and does not necessarily represent the official views of the NIH.

## Conflict of interest

The authors declare that the research was conducted in the absence of any commercial or financial relationships that could be construed as a potential conflict of interest.

## Publisher’s note

All claims expressed in this article are solely those of the authors and do not necessarily represent those of their affiliated organizations, or those of the publisher, the editors and the reviewers. Any product that may be evaluated in this article, or claim that may be made by its manufacturer, is not guaranteed or endorsed by the publisher.

## Footnotes

^1^ https://www.ncbi.nlm.nih.gov/tools/cobalt/re_cobalt.cgi

^2^ https://psort.hgc.jp/form2.html

^3^ http://pridb.gdcb.iastate.edu/RPISeq/
