# Supplementary material for: Nuclear translocation of spike mRNA and protein is a novel feature of SARS-CoV-2
Source: Front Microbiol. 2023 Jan 26;14:1073789. doi: 10.3389/fmicb.2023.1073789 (PMC9909199; doi:10.3389/fmicb.2023.1073789)
Supplement: Supplementary file 2 [file Data_Sheet_2.PDF]

RNA-Protein Interaction Prediction (RPISeq)

Dobbs and Honavar Laboratories

Input Sequences

Protein:
MSDNGPQNQRNAPRITFGGPSDSTGSNQNGERSGARSKQRRPQGLPNNTASWFTALTQHG
KEDLKFPRGQGVPINTNSSPDDQIGYYRRATRRIRGGDGKMKDLSPRWYFYYLGTGPEAG
LPYGANKDGIIWVATEGALNTPKDHIGTRNPANNAAIVLQLPQGTTLPKGFYAEGSRGGS
QASSRSSSRSRNSSRNSTPGSSRGTSPARMAGNGGDAALALLLLDRLNQLESKMSGKGQQ
QQGQTVTKKSAAEASKKPRQKRTATKAYNVTQAFGRRGPEQTQGNFGDQELIRQGTDYKH
WPQIAQFAPSASAFFGMSRIGMEVTPSGTWLTYTGAIKLDDKDPNFKDQVILLNKHIDAY
KTFPPTEPKKDKKKKADETQALPQRQKKQQTVTLLPAADLDDFSKQLQQSMSSADSTQA

RNA:
AUUAAAGGUUUAUACCUUCCCAGGUAACAAACCAACCAACUUUCGAUCUCUUGUAGAUCU
GUUCUCUAAACGAACUUUAAAAUCUGUGUGGCUGUCACUCGGCUGCAUGCUUAGUGCACU
CACGCAGUAUAAUUAAUAACUAAUUACUGUCGUUGACAGGACACGAGUAACUCGUCUAUC
UUCUGCAGGCUGCUUACGGUUUCGUCCGUGUUGCAGCCGAUCAUCAGCACAUCUAGGUUU
CGUCCGGGUGUGACCGAAAGGUAAGAUGGAGAGCCUUGUCCCUGGUUUCAACGAGAAAAC
ACACGUCCAACUCAGUUUGCCUGUUUUACAGGUUCGCGACGUGCUCGUACGUGGCUUUGG
AGACUCCGUGGAGGAGGUCUUAUCAGAGGCACGUCAACAUCUUAAAGAUGGCACUUGUGG
CUUAGUAGAAGUUGAAAAAGGCGUUUUGCCUCAACUUGAACAGCCCUAUGUGUUCAUCAA
ACGUUCGGAUGCUCGAACUGCACCUCAUGGUCAUGUUAUGGUUGAGCUGGUAGCAGAACU
CGAAGGCAUUCAGUACGGUCGUAGUGGUGAGACACUUGGUGUCCUUGUCCCUCAUGUGGG
CGAAAUACCAGUGGCUUACCGCAAGGUUCUUCUUCGUAAGAACGGUAAUAAAGGAGCUGG
UGGCCAUAGUUACGGCGCCGAUCUAAAGUCAUUUGACUUAGGCGACGAGCUUGGCACUGA
UCCUUAUGAAGAUUUUCAAGAAAACUGGAACACUAAACAUAGCAGUGGUGUUACCCGUGA
ACUCAUGCGUGAGCUUAACGGAGGGGCAUACACUCGCUAUGUCGAUAACAACUUCUGUGG
CCCUGAUGGCUACCCUCUUGAGUGCAUUAAAGACCUUCUAGCACGUGCUGGUAAAGCUUC
AUGCACUUUGUCCGAACAACUGGACUUUAUUGACACUAAGAGGGGUGUAUACUGCUGCCG
UGAACAUGAGCAUGAAAUUGCUUGGUACACGGAACGUUCUGAAAAGAGCUAUGAAUUGCA
GACACCUUUUGAAAUUAAAUUGGCAAAGAAAUUUGACACCUUCAAUGGGGAAUGUCCAAA
UUUUGUAUUUCCCUUAAAUUCCAUAAUCAAGACUAUUCAACCAAGGGUUGAAAAGAAAAA
GCUUGAUGGCUUUAUGGGUAGAAUUCGAUCUGUCUAUCCAGUUGCGUCACCAAAUGAAUG
CAACCAAAUGUGCCUUUCAACUCUCAUGAAGUGUGAUCAUUGUGGUGAAACUUCAUGGCA
GACGGGCGAUUUUGUUAAAGCCACUUGCGAAUUUUGUGGCACUGAGAAUUUGACUAAAGA
AGGUGCCACUACUUGUGGUUACUUACCCCAAAAUGCUGUUGUUAAAAUUUAUUGUCCAGC
AUGUCACAAUUCAGAAGUAGGACCUGAGCAUAGUCUUGCCGAAUACCAUAAUGAAUCUGG
CUUGAAAACCAUUCUUCGUAAGGGUGGUCGCACUAUUGCCUUUGGAGGCUGUGUGUUCUC
UUAUGUUGGUUGCCAUAACAAGUGUGCCUAUUGGGUUCCACGUGCUAGCGCUAACAUAGG
UUGUAACCAUACAGGUGUUGUUGGAGAAGGUUCCGAAGGUCUUAAUGACAACCUUCUUGA
AAUACUCCAAAAAGAGAAAGUCAACAUCAAUAUUGUUGGUGACUUUAAACUUAAUGAAGA
GAUCGCCAUUAUUUUGGCAUCUUUUUCUGCUUCCACAAGUGCUUUUGUGGAAACUGUGAA
AGGUUUGGAUUAUAAAGCAUUCAAACAAAUUGUUGAAUCCUGUGGUAAUUUUAAAGUUAC
AAAAGGAAAAGCUAAAAAAGGUGCCUGGAAUAUUGGUGAACAGAAAUCAAUACUGAGUCC
UCUUUAUGCAUUUGCAUCAGAGGCUGCUCGUGUUGUACGAUCAAUUUUCUCCCGCACUCU
UGAAACUGCUCAAAAUUCUGUGCGUGUUUUACAGAAGGCCGCUAUAACAAUACUAGAUGG
AAUUUCACAGUAUUCACUGAGACUCAUUGAUGCUAUGAUGUUCACAUCUGAUUUGGCUAC
UAACAAUCUAGUUGUAAUGGCCUACAUUACAGGUGGUGUUGUUCAGUUGACUUCGCAGUG
GCUAACUAACAUCUUUGGCACUGUUUAUGAAAAACUCAAACCCGUCCUUGAUUGGCUUGA
AGAGAAGUUUAAGGAAGGUGUAGAGUUUCUUAGAGACGGUUGGGAAAUUGUUAAAUUUAU
CUCAACCUGUGCUUGUGAAAUUGUCGGUGGACAAAUUGUCACCUGUGCAAAGGAAAUUAA
GGAGAGUGUUCAGACAUUCUUUAAGCUUGUAAAUAAAUUUUUGGCUUUGUGUGCUGACUC
UAUCAUUAUUGGUGGAGCUAAACUUAAAGCCUUGAAUUUAGGUGAAACAUUUGUCACGCA
CUCAAAGGGAUUGUACAGAAAGUGUGUUAAAUCCAGAGAAGAAACUGGCCUACUCAUGCC
UCUAAAAGCCCCAAAAGAAAUUAUCUUCUUAGAGGGAGAAACACUUCCCACAGAAGUGUU
AACAGAGGAAGUUGUCUUGAAAACUGGUGAUUUACAACCAUUAGAACAACCUACUAGUGA
AGCUGUUGAAGCUCCAUUGGUUGGUACACCAGUUUGUAUUAACGGGCUUAUGUUGCUCGA
AAUCAAAGACACAGAAAAGUACUGUGCCCUUGCACCUAAUAUGAUGGUAACAAACAAUAC
CUUCACACUCAAAGGCGGUGCACCAACAAAGGUUACUUUUGGUGAUGACACUGUGAUAGA
AGUGCAAGGUUACAAGAGUGUGAAUAUCACUUUUGAACUUGAUGAAAGGAUUGAUAAAGU
ACUUAAUGAGAAGUGCUCUGCCUAUACAGUUGAACUCGGUACAGAAGUAAAUGAGUUCGC
CUGUGUUGUGGCAGAUGCUGUCAUAAAAACUUUGCAACCAGUAUCUGAAUUACUUACACC
ACUGGGCAUUGAUUUAGAUGAGUGGAGUAUGGCUACAUACUACUUAUUUGAUGAGUCUGG

UGAGUUUAAAUUGGCUUCACAUAUGUAUUGUUCUUUCUACCCUCCAGAUGAGGAUGAAGA
AGAAGGUGAUUGUGAAGAAGAAGAGUUUGAGCCAUCAACUCAAUAUGAGUAUGGUACUGA
AGAUGAUUACCAAGGUAAACCUUUGGAAUUUGGUGCCACUUCUGCUGCUCUUCAACCUGA
AGAAGAGCAAGAAGAAGAUUGGUUAGAUGAUGAUAGUCAACAAACUGUUGGUCAACAAGA
CGGCAGUGAGGACAAUCAGACAACUACUAUUCAAACAAUUGUUGAGGUUCAACCUCAAUU
AGAGAUGGAACUUACACCAGUUGUUCAGACUAUUGAAGUGAAUAGUUUUAGUGGUUAUUU
AAAACUUACUGACAAUGUAUACAUUAAAAAUGCAGACAUUGUGGAAGAAGCUAAAAAGGU
AAAACCAACAGUGGUUGUUAAUGCAGCCAAUGUUUACCUUAAACAUGGAGGAGGUGUUGC
AGGAGCCUUAAAUAAGGCUACUAACAAUGCCAUGCAAGUUGAAUCUGAUGAUUACAUAGC
UACUAAUGGACCACUUAAAGUGGGUGGUAGUUGUGUUUUAAGCGGACACAAUCUUGCUAA
ACACUGUCUUCAUGUUGUCGGCCCAAAUGUUAACAAAGGUGAAGACAUUCAACUUCUUAA
GAGUGCUUAUGAAAAUUUUAAUCAGCACGAAGUUCUACUUGCACCAUUAUUAUCAGCUGG
UAUUUUUGGUGCUGACCCUAUACAUUCUUUAAGAGUUUGUGUAGAUACUGUUCGCACAAA
UGUCUACUUAGCUGUCUUUGAUAAAAAUCUCUAUGACAAACUUGUUUCAAGCUUUUUGGA
AAUGAAGAGUGAAAAGCAAGUUGAACAAAAGAUCGCUGAGAUUCCUAAAGAGGAAGUUAA
GCCAUUUAUAACUGAAAGUAAACCUUCAGUUGAACAGAGAAAACAAGAUGAUAAGAAAAU
CAAAGCUUGUGUUGAAGAAGUUACAACAACUCUGGAAGAAACUAAGUUCCUCACAGAAAA
CUUGUUACUUUAUAUUGACAUUAAUGGCAAUCUUCAUCCAGAUUCUGCCACUCUUGUUAG
UGACAUUGACAUCACUUUCUUAAAGAAAGAUGCUCCAUAUAUAGUGGGUGAUGUUGUUCA
AGAGGGUGUUUUAACUGCUGUGGUUAUACCUACUAAAAAGGCUGGUGGCACUACUGAAAU
GCUAGCGAAAGCUUUGAGAAAAGUGCCAACAGACAAUUAUAUAACCACUUACCCGGGUCA
GGGUUUAAAUGGUUACACUGUAGAGGAGGCAAAGACAGUGCUUAAAAAGUGUAAAAGUGC
CUUUUACAUUCUACCAUCUAUUAUCUCUAAUGAGAAGCAAGAAAUUCUUGGAACUGUUUC
UUGGAAUUUGCGAGAAAUGCUUGCACAUGCAGAAGAAACACGCAAAUUAAUGCCUGUCUG
UGUGGAAACUAAAGCCAUAGUUUCAACUAUACAGCGUAAAUAUAAGGGUAUUAAAAUACA
AGAGGGUGUGGUUGAUUAUGGUGCUAGAUUUUACUUUUACACCAGUAAAACAACUGUAGC
GUCACUUAUCAACACACUUAACGAUCUAAAUGAAACUCUUGUUACAAUGCCACUUGGCUA
UGUAACACAUGGCUUAAAUUUGGAAGAAGCUGCUCGGUAUAUGAGAUCUCUCAAAGUGCC
AGCUACAGUUUCUGUUUCUUCACCUGAUGCUGUUACAGCGUAUAAUGGUUAUCUUACUUC
UUCUUCUAAAACACCUGAAGAACAUUUUAUUGAAACCAUCUCACUUGCUGGUUCCUAUAA
AGAUUGGUCCUAUUCUGGACAAUCUACACAACUAGGUAUAGAAUUUCUUAAGAGAGGUGA
UAAAAGUGUAUAUUACACUAGUAAUCCUACCACAUUCCACCUAGAUGGUGAAGUUAUCAC
CUUUGACAAUCUUAAGACACUUCUUUCUUUGAGAGAAGUGAGGACUAUUAAGGUGUUUAC
AACAGUAGACAACAUUAACCUCCACACGCAAGUUGUGGACAUGUCAAUGACAUAUGGACA
ACAGUUUGGUCCAACUUAUUUGGAUGGAGCUGAUGUUACUAAAAUAAAACCUCAUAAUUC
ACAUGAAGGUAAAACAUUUUAUGUUUUACCUAAUGAUGACACUCUACGUGUUGAGGCUUU
UGAGUACUACCACACAACUGAUCCUAGUUUUCUGGGUAGGUACAUGUCAGCAUUAAAUCA
CACUAAAAAGUGGAAAUACCCACAAGUUAAUGGUUUAACUUCUAUUAAAUGGGCAGAUAA
CAACUGUUAUCUUGCCACUGCAUUGUUAACACUCCAACAAAUAGAGUUGAAGUUUAAUCC
ACCUGCUCUACAAGAUGCUUAUUACAGAGCAAGGGCUGGUGAAGCUGCUAACUUUUGUGC
ACUUAUCUUAGCCUACUGUAAUAAGACAGUAGGUGAGUUAGGUGAUGUUAGAGAAACAAU
GAGUUACUUGUUUCAACAUGCCAAUUUAGAUUCUUGCAAAAGAGUCUUGAACGUGGUGUG
UAAAACUUGUGGACAACAGCAGACAACCCUUAAGGGUGUAGAAGCUGUUAUGUACAUGGG
CACACUUUCUUAUGAACAAUUUAAGAAAGGUGUUCAGAUACCUUGUACGUGUGGUAAACA
AGCUACAAAAUAUCUAGUACAACAGGAGUCACCUUUUGUUAUGAUGUCAGCACCACCUGC
UCAGUAUGAACUUAAGCAUGGUACAUUUACUUGUGCUAGUGAGUACACUGGUAAUUACCA
GUGUGGUCACUAUAAACAUAUAACUUCUAAAGAAACUUUGUAUUGCAUAGACGGUGCUUU
ACUUACAAAGUCCUCAGAAUACAAAGGUCCUAUUACGGAUGUUUUCUACAAAGAAAACAG
UUACACAACAACCAUAAAACCAGUUACUUAUAAAUUGGAUGGUGUUGUUUGUACAGAAAU
UGACCCUAAGUUGGACAAUUAUUAUAAGAAAGACAAUUCUUAUUUCACAGAGCAACCAAU
UGAUCUUGUACCAAACCAACCAUAUCCAAACGCAAGCUUCGAUAAUUUUAAGUUUGUAUG
UGAUAAUAUCAAAUUUGCUGAUGAUUUAAACCAGUUAACUGGUUAUAAGAAACCUGCUUC
AAGAGAGCUUAAAGUUACAUUUUUCCCUGACUUAAAUGGUGAUGUGGUGGCUAUUGAUUA
UAAACACUACACACCCUCUUUUAAGAAAGGAGCUAAAUUGUUACAUAAACCUAUUGUUUG
GCAUGUUAACAAUGCAACUAAUAAAGCCACGUAUAAACCAAAUACCUGGUGUAUACGUUG
UCUUUGGAGCACAAAACCAGUUGAAACAUCAAAUUCGUUUGAUGUACUGAAGUCAGAGGA
CGCGCAGGGAAUGGAUAAUCUUGCCUGCGAAGAUCUAAAACCAGUCUCUGAAGAAGUAGU
GGAAAAUCCUACCAUACAGAAAGACGUUCUUGAGUGUAAUGUGAAAACUACCGAAGUUGU
AGGAGACAUUAUACUUAAACCAGCAAAUAAUAGUUUAAAAAUUACAGAAGAGGUUGGCCA
CACAGAUCUAAUGGCUGCUUAUGUAGACAAUUCUAGUCUUACUAUUAAGAAACCUAAUGA
AUUAUCUAGAGUAUUAGGUUUGAAAACCCUUGCUACUCAUGGUUUAGCUGCUGUUAAUAG
UGUCCCUUGGGAUACUAUAGCUAAUUAUGCUAAGCCUUUUCUUAACAAAGUUGUUAGUAC
AACUACUAACAUAGUUACACGGUGUUUAAACCGUGUUUGUACUAAUUAUAUGCCUUAUUU
CUUUACUUUAUUGCUACAAUUGUGUACUUUUACUAGAAGUACAAAUUCUAGAAUUAAAGC
AUCUAUGCCGACUACUAUAGCAAAGAAUACUGUUAAGAGUGUCGGUAAAUUUUGUCUAGA
GGCUUCAUUUAAUUAUUUGAAGUCACCUAAUUUUUCUAAACUGAUAAAUAUUAUAAUUUG
GUUUUUACUAUUAAGUGUUUGCCUAGGUUCUUUAAUCUACUCAACCGCUGCUUUAGGUGU
UUUAAUGUCUAAUUUAGGCAUGCCUUCUUACUGUACUGGUUACAGAGAAGGCUAUUUGAA

CUCUACUAAUGUCACUAUUGCAACCUACUGUACUGGUUCUAUACCUUGUAGUGUUUGUCU
UAGUGGUUUAGAUUCUUUAGACACCUAUCCUUCUUUAGAAACUAUACAAAUUACCAUUUC
AUCUUUUAAAUGGGAUUUAACUGCUUUUGGCUUAGUUGCAGAGUGGUUUUUGGCAUAUAU
UCUUUUCACUAGGUUUUUCUAUGUACUUGGAUUGGCUGCAAUCAUGCAAUUGUUUUUCAG
CUAUUUUGCAGUACAUUUUAUUAGUAAUUCUUGGCUUAUGUGGUUAAUAAUUAAUCUUGU
ACAAAUGGCCCCGAUUUCAGCUAUGGUUAGAAUGUACAUCUUCUUUGCAUCAUUUUAUUA
UGUAUGGAAAAGUUAUGUGCAUGUUGUAGACGGUUGUAAUUCAUCAACUUGUAUGAUGUG
UUACAAACGUAAUAGAGCAACAAGAGUCGAAUGUACAACUAUUGUUAAUGGUGUUAGAAG
GUCCUUUUAUGUCUAUGCUAAUGGAGGUAAAGGCUUUUGCAAACUACACAAUUGGAAUUG
UGUUAAUUGUGAUACAUUCUGUGCUGGUAGUACAUUUAUUAGUGAUGAAGUUGCGAGAGA
CUUGUCACUACAGUUUAAAAGACCAAUAAAUCCUACUGACCAGUCUUCUUACAUCGUUGA
UAGUGUUACAGUGAAGAAUGGUUCCAUCCAUCUUUACUUUGAUAAAGCUGGUCAAAAGAC
UUAUGAAAGACAUUCUCUCUCUCAUUUUGUUAACUUAGACAACCUGAGAGCUAAUAACAC
UAAAGGUUCAUUGCCUAUUAAUGUUAUAGUUUUUGAUGGUAAAUCAAAAUGUGAAGAAUC
AUCUGCAAAAUCAGCGUCUGUUUACUACAGUCAGCUUAUGUGUCAACCUAUACUGUUACU
AGAUCAGGCAUUAGUGUCUGAUGUUGGUGAUAGUGCGGAAGUUGCAGUUAAAAUGUUUGA
UGCUUACGUUAAUACGUUUUCAUCAACUUUUAACGUACCAAUGGAAAAACUCAAAACACU
AGUUGCAACUGCAGAAGCUGAACUUGCAAAGAAUGUGUCCUUAGACAAUGUCUUAUCUAC
UUUUAUUUCAGCAGCUCGGCAAGGGUUUGUUGAUUCAGAUGUAGAAACUAAAGAUGUUGU
UGAAUGUCUUAAAUUGUCACAUCAAUCUGACAUAGAAGUUACUGGCGAUAGUUGUAAUAA
CUAUAUGCUCACCUAUAACAAAGUUGAAAACAUGACACCCCGUGACCUUGGUGCUUGUAU
UGACUGUAGUGCGCGUCAUAUUAAUGCGCAGGUAGCAAAAAGUCACAACAUUGCUUUGAU
AUGGAACGUUAAAGAUUUCAUGUCAUUGUCUGAACAACUACGAAAACAAAUACGUAGUGC
UGCUAAAAAGAAUAACUUACCUUUUAAGUUGACAUGUGCAACUACUAGACAAGUUGUUAA
UGUUGUAACAACAAAGAUAGCACUUAAGGGUGGUAAAAUUGUUAAUAAUUGGUUGAAGCA
GUUAAUUAAAGUUACACUUGUGUUCCUUUUUGUUGCUGCUAUUUUCUAUUUAAUAACACC
UGUUCAUGUCAUGUCUAAACAUACUGACUUUUCAAGUGAAAUCAUAGGAUACAAGGCUAU
UGAUGGUGGUGUCACUCGUGACAUAGCAUCUACAGAUACUUGUUUUGCUAACAAACAUGC
UGAUUUUGACACAUGGUUUAGUCAGCGUGGUGGUAGUUAUACUAAUGACAAAGCUUGCCC
AUUGAUUGCUGCAGUCAUAACAAGAGAAGUGGGUUUUGUCGUGCCUGGUUUGCCUGGCAC
GAUAUUACGCACAACUAAUGGUGACUUUUUGCAUUUCUUACCUAGAGUUUUUAGUGCAGU
UGGUAACAUCUGUUACACACCAUCAAAACUUAUAGAGUACACUGACUUUGCAACAUCAGC
UUGUGUUUUGGCUGCUGAAUGUACAAUUUUUAAAGAUGCUUCUGGUAAGCCAGUACCAUA
UUGUUAUGAUACCAAUGUACUAGAAGGUUCUGUUGCUUAUGAAAGUUUACGCCCUGACAC
ACGUUAUGUGCUCAUGGAUGGCUCUAUUAUUCAAUUUCCUAACACCUACCUUGAAGGUUC
UGUUAGAGUGGUAACAACUUUUGAUUCUGAGUACUGUAGGCACGGCACUUGUGAAAGAUC
AGAAGCUGGUGUUUGUGUAUCUACUAGUGGUAGAUGGGUACUUAACAAUGAUUAUUACAG
AUCUUUACCAGGAGUUUUCUGUGGUGUAGAUGCUGUAAAUUUACUUACUAAUAUGUUUAC
ACCACUAAUUCAACCUAUUGGUGCUUUGGACAUAUCAGCAUCUAUAGUAGCUGGUGGUAU
UGUAGCUAUCGUAGUAACAUGCCUUGCCUACUAUUUUAUGAGGUUUAGAAGAGCUUUUGG
UGAAUACAGUCAUGUAGUUGCCUUUAAUACUUUACUAUUCCUUAUGUCAUUCACUGUACU
CUGUUUAACACCAGUUUACUCAUUCUUACCUGGUGUUUAUUCUGUUAUUUACUUGUACUU
GACAUUUUAUCUUACUAAUGAUGUUUCUUUUUUAGCACAUAUUCAGUGGAUGGUUAUGUU
CACACCUUUAGUACCUUUCUGGAUAACAAUUGCUUAUAUCAUUUGUAUUUCCACAAAGCA
UUUCUAUUGGUUCUUUAGUAAUUACCUAAAGAGACGUGUAGUCUUUAAUGGUGUUUCCUU
UAGUACUUUUGAAGAAGCUGCGCUGUGCACCUUUUUGUUAAAUAAAGAAAUGUAUCUAAA
GUUGCGUAGUGAUGUGCUAUUACCUCUUACGCAAUAUAAUAGAUACUUAGCUCUUUAUAA
UAAGUACAAGUAUUUUAGUGGAGCAAUGGAUACAACUAGCUACAGAGAAGCUGCUUGUUG
UCAUCUCGCAAAGGCUCUCAAUGACUUCAGUAACUCAGGUUCUGAUGUUCUUUACCAACC
ACCACAAACCUCUAUCACCUCAGCUGUUUUGCAGAGUGGUUUUAGAAAAAUGGCAUUCCC
AUCUGGUAAAGUUGAGGGUUGUAUGGUACAAGUAACUUGUGGUACAACUACACUUAACGG
UCUUUGGCUUGAUGACGUAGUUUACUGUCCAAGACAUGUGAUCUGCACCUCUGAAGACAU
GCUUAACCCUAAUUAUGAAGAUUUACUCAUUCGUAAGUCUAAUCAUAAUUUCUUGGUACA
GGCUGGUAAUGUUCAACUCAGGGUUAUUGGACAUUCUAUGCAAAAUUGUGUACUUAAGCU
UAAGGUUGAUACAGCCAAUCCUAAGACACCUAAGUAUAAGUUUGUUCGCAUUCAACCAGG
ACAGACUUUUUCAGUGUUAGCUUGUUACAAUGGUUCACCAUCUGGUGUUUACCAAUGUGC
UAUGAGGCCCAAUUUCACUAUUAAGGGUUCAUUCCUUAAUGGUUCAUGUGGUAGUGUUGG
UUUUAACAUAGAUUAUGACUGUGUCUCUUUUUGUUACAUGCACCAUAUGGAAUUACCAAC
UGGAGUUCAUGCUGGCACAGACUUAGAAGGUAACUUUUAUGGACCUUUUGUUGACAGGCA
AACAGCACAAGCAGCUGGUACGGACACAACUAUUACAGUUAAUGUUUUAGCUUGGUUGUA
CGCUGCUGUUAUAAAUGGAGACAGGUGGUUUCUCAAUCGAUUUACCACAACUCUUAAUGA
CUUUAACCUUGUGGCUAUGAAGUACAAUUAUGAACCUCUAACACAAGACCAUGUUGACAU
ACUAGGACCUCUUUCUGCUCAAACUGGAAUUGCCGUUUUAGAUAUGUGUGCUUCAUUAAA
AGAAUUACUGCAAAAUGGUAUGAAUGGACGUACCAUAUUGGGUAGUGCUUUAUUAGAAGA
UGAAUUUACACCUUUUGAUGUUGUUAGACAAUGCUCAGGUGUUACUUUCCAAAGUGCAGU
GAAAAGAACAAUCAAGGGUACACACCACUGGUUGUUACUCACAAUUUUGACUUCACUUUU
AGUUUUAGUCCAGAGUACUCAAUGGUCUUUGUUCUUUUUUUUGUAUGAAAAUGCCUUUUU
ACCUUUUGCUAUGGGUAUUAUUGCUAUGUCUGCUUUUGCAAUGAUGUUUGUCAAACAUAA

GCAUGCAUUUCUCUGUUUGUUUUUGUUACCUUCUCUUGCCACUGUAGCUUAUUUUAAUAU
GGUCUAUAUGCCUGCUAGUUGGGUGAUGCGUAUUAUGACAUGGUUGGAUAUGGUUGAUAC
UAGUUUGUCUGGUUUUAAGCUAAAAGACUGUGUUAUGUAUGCAUCAGCUGUAGUGUUACU
AAUCCUUAUGACAGCAAGAACUGUGUAUGAUGAUGGUGCUAGGAGAGUGUGGACACUUAU
GAAUGUCUUGACACUCGUUUAUAAAGUUUAUUAUGGUAAUGCUUUAGAUCAAGCCAUUUC
CAUGUGGGCUCUUAUAAUCUCUGUUACUUCUAACUACUCAGGUGUAGUUACAACUGUCAU
GUUUUUGGCCAGAGGUAUUGUUUUUAUGUGUGUUGAGUAUUGCCCUAUUUUCUUCAUAAC
UGGUAAUACACUUCAGUGUAUAAUGCUAGUUUAUUGUUUCUUAGGCUAUUUUUGUACUUG
UUACUUUGGCCUCUUUUGUUUACUCAACCGCUACUUUAGACUGACUCUUGGUGUUUAUGA
UUACUUAGUUUCUACACAGGAGUUUAGAUAUAUGAAUUCACAGGGACUACUCCCACCCAA
GAAUAGCAUAGAUGCCUUCAAACUCAACAUUAAAUUGUUGGGUGUUGGUGGCAAACCUUG
UAUCAAAGUAGCCACUGUACAGUCUAAAAUGUCAGAUGUAAAGUGCACAUCAGUAGUCUU
ACUCUCAGUUUUGCAACAACUCAGAGUAGAAUCAUCAUCUAAAUUGUGGGCUCAAUGUGU
CCAGUUACACAAUGACAUUCUCUUAGCUAAAGAUACUACUGAAGCCUUUGAAAAAAUGGU
UUCACUACUUUCUGUUUUGCUUUCCAUGCAGGGUGCUGUAGACAUAAACAAGCUUUGUGA
AGAAAUGCUGGACAACAGGGCAACCUUACAAGCUAUAGCCUCAGAGUUUAGUUCCCUUCC
AUCAUAUGCAGCUUUUGCUACUGCUCAAGAAGCUUAUGAGCAGGCUGUUGCUAAUGGUGA
UUCUGAAGUUGUUCUUAAAAAGUUGAAGAAGUCUUUGAAUGUGGCUAAAUCUGAAUUUGA
CCGUGAUGCAGCCAUGCAACGUAAGUUGGAAAAGAUGGCUGAUCAAGCUAUGACCCAAAU
GUAUAAACAGGCUAGAUCUGAGGACAAGAGGGCAAAAGUUACUAGUGCUAUGCAGACAAU
GCUUUUCACUAUGCUUAGAAAGUUGGAUAAUGAUGCACUCAACAACAUUAUCAACAAUGC
AAGAGAUGGUUGUGUUCCCUUGAACAUAAUACCUCUUACAACAGCAGCCAAACUAAUGGU
UGUCAUACCAGACUAUAACACAUAUAAAAAUACGUGUGAUGGUACAACAUUUACUUAUGC
AUCAGCAUUGUGGGAAAUCCAACAGGUUGUAGAUGCAGAUAGUAAAAUUGUUCAACUUAG
UGAAAUUAGUAUGGACAAUUCACCUAAUUUAGCAUGGCCUCUUAUUGUAACAGCUUUAAG
GGCCAAUUCUGCUGUCAAAUUACAGAAUAAUGAGCUUAGUCCUGUUGCACUACGACAGAU
GUCUUGUGCUGCCGGUACUACACAAACUGCUUGCACUGAUGACAAUGCGUUAGCUUACUA
CAACACAACAAAGGGAGGUAGGUUUGUACUUGCACUGUUAUCCGAUUUACAGGAUUUGAA
AUGGGCUAGAUUCCCUAAGAGUGAUGGAACUGGUACUAUCUAUACAGAACUGGAACCACC
UUGUAGGUUUGUUACAGACACACCUAAAGGUCCUAAAGUGAAGUAUUUAUACUUUAUUAA
AGGAUUAAACAACCUAAAUAGAGGUAUGGUACUUGGUAGUUUAGCUGCCACAGUACGUCU
ACAAGCUGGUAAUGCAACAGAAGUGCCUGCCAAUUCAACUGUAUUAUCUUUCUGUGCUUU
UGCUGUAGAUGCUGCUAAAGCUUACAAAGAUUAUCUAGCUAGUGGGGGACAACCAAUCAC
UAAUUGUGUUAAGAUGUUGUGUACACACACUGGUACUGGUCAGGCAAUAACAGUUACACC
GGAAGCCAAUAUGGAUCAAGAAUCCUUUGGUGGUGCAUCGUGUUGUCUGUACUGCCGUUG
CCACAUAGAUCAUCCAAAUCCUAAAGGAUUUUGUGACUUAAAAGGUAAGUAUGUACAAAU
ACCUACAACUUGUGCUAAUGACCCUGUGGGUUUUACACUUAAAAACACAGUCUGUACCGU
CUGCGGUAUGUGGAAAGGUUAUGGCUGUAGUUGUGAUCAACUCCGCGAACCCAUGCUUCA
GUCAGCUGAUGCACAAUCGUUUUUAAACGGGUUUGCGGUGUAAGUGCAGCCCGUCUUACA
CCGUGCGGCACAGGCACUAGUACUGAUGUCGUAUACAGGGCUUUUGACAUCUACAAUGAU
AAAGUAGCUGGUUUUGCUAAAUUCCUAAAAACUAAUUGUUGUCGCUUCCAAGAAAAGGAC
GAAGAUGACAAUUUAAUUGAUUCUUACUUUGUAGUUAAGAGACACACUUUCUCUAACUAC
CAACAUGAAGAAACAAUUUAUAAUUUACUUAAGGAUUGUCCAGCUGUUGCUAAACAUGAC
UUCUUUAAGUUUAGAAUAGACGGUGACAUGGUACCACAUAUAUCACGUCAACGUCUUACU
AAAUACACAAUGGCAGACCUCGUCUAUGCUUUAAGGCAUUUUGAUGAAGGUAAUUGUGAC
ACAUUAAAAGAAAUACUUGUCACAUACAAUUGUUGUGAUGAUGAUUAUUUCAAUAAAAAG
GACUGGUAUGAUUUUGUAGAAAACCCAGAUAUAUUACGCGUAUACGCCAACUUAGGUGAA
CGUGUACGCCAAGCUUUGUUAAAAACAGUACAAUUCUGUGAUGCCAUGCGAAAUGCUGGU
AUUGUUGGUGUACUGACAUUAGAUAAUCAAGAUCUCAAUGGUAACUGGUAUGAUUUCGGU
GAUUUCAUACAAACCACGCCAGGUAGUGGAGUUCCUGUUGUAGAUUCUUAUUAUUCAUUG
UUAAUGCCUAUAUUAACCUUGACCAGGGCUUUAACUGCAGAGUCACAUGUUGACACUGAC
UUAACAAAGCCUUACAUUAAGUGGGAUUUGUUAAAAUAUGACUUCACGGAAGAGAGGUUA
AAACUCUUUGACCGUUAUUUUAAAUAUUGGGAUCAGACAUACCACCCAAAUUGUGUUAAC
UGUUUGGAUGACAGAUGCAUUCUGCAUUGUGCAAACUUUAAUGUUUUAUUCUCUACAGUG
UUCCCACCUACAAGUUUUGGACCACUAGUGAGAAAAAUAUUUGUUGAUGGUGUUCCAUUU
GUAGUUUCAACUGGAUACCACUUCAGAGAGCUAGGUGUUGUACAUAAUCAGGAUGUAAAC
UUACAUAGCUCUAGACUUAGUUUUAAGGAAUUACUUGUGUAUGCUGCUGACCCUGCUAUG
CACGCUGCUUCUGGUAAUCUAUUACUAGAUAAACGCACUACGUGCUUUUCAGUAGCUGCA
CUUACUAACAAUGUUGCUUUUCAAACUGUCAAACCCGGUAAUUUUAACAAAGACUUCUAU
GACUUUGCUGUGUCUAAGGGUUUCUUUAAGGAAGGAAGUUCUGUUGAAUUAAAACACUUC
UUCUUUGCUCAGGAUGGUAAUGCUGCUAUCAGCGAUUAUGACUACUAUCGUUAUAAUCUA
CCAACAAUGUGUGAUAUCAGACAACUACUAUUUGUAGUUGAAGUUGUUGAUAAGUACUUU
GAUUGUUACGAUGGUGGCUGUAUUAAUGCUAACCAAGUCAUCGUCAACAACCUAGACAAA
UCAGCUGGUUUUCCAUUUAAUAAAUGGGGUAAGGCUAGACUUUAUUAUGAUUCAAUGAGU
UAUGAGGAUCAAGAUGCACUUUUCGCAUAUACAAAACGUAAUGUCAUCCCUACUAUAACU
CAAAUGAAUCUUAAGUAUGCCAUUAGUGCAAAGAAUAGAGCUCGCACCGUAGCUGGUGUC
UCUAUCUGUAGUACUAUGACCAAUAGACAGUUUCAUCAAAAAUUAUUGAAAUCAAUAGCC
GCCACUAGAGGAGCUACUGUAGUAAUUGGAACAAGCAAAUUCUAUGGUGGUUGGCACAAC

AUGUUAAAAACUGUUUAUAGUGAUGUAGAAAACCCUCACCUUAUGGGUUGGGAUUAUCCU
AAAUGUGAUAGAGCCAUGCCUAACAUGCUUAGAAUUAUGGCCUCACUUGUUCUUGCUCGC
AAACAUACAACGUGUUGUAGCUUGUCACACCGUUUCUAUAGAUUAGCUAAUGAGUGUGCU
CAAGUAUUGAGUGAAAUGGUCAUGUGUGGCGGUUCACUAUAUGUUAAACCAGGUGGAACC
UCAUCAGGAGAUGCCACAACUGCUUAUGCUAAUAGUGUUUUUAACAUUUGUCAAGCUGUC
ACGGCCAAUGUUAAUGCACUUUUAUCUACUGAUGGUAACAAAAUUGCCGAUAAGUAUGUC
CGCAAUUUACAACACAGACUUUAUGAGUGUCUCUAUAGAAAUAGAGAUGUUGACACAGAC
UUUGUGAAUGAGUUUUACGCAUAUUUGCGUAAACAUUUCUCAAUGAUGAUACUCUCUGAC
GAUGCUGUUGUGUGUUUCAAUAGCACUUAUGCAUCUCAAGGUCUAGUGGCUAGCAUAAAG
AACUUUAAGUCAGUUCUUUAUUAUCAAAACAAUGUUUUUAUGUCUGAAGCAAAAUGUUGG
ACUGAGACUGACCUUACUAAAGGACCUCAUGAAUUUUGCUCUCAACAUACAAUGCUAGUU
AAACAGGGUGAUGAUUAUGUGUACCUUCCUUACCCAGAUCCAUCAAGAAUCCUAGGGGCC
GGCUGUUUUGUAGAUGAUAUCGUAAAAACAGAUGGUACACUUAUGAUUGAACGGUUCGUG
UCUUUAGCUAUAGAUGCUUACCCACUUACUAAACAUCCUAAUCAGGAGUAUGCUGAUGUC
UUUCAUUUGUACUUACAAUACAUAAGAAAGCUACAUGAUGAGUUAACAGGACACAUGUUA
GACAUGUAUUCUGUUAUGCUUACUAAUGAUAACACUUCAAGGUAUUGGGAACCUGAGUUU
UAUGAGGCUAUGUACACACCGCAUACAGUCUUACAGGCUGUUGGGGCUUGUGUUCUUUGC
AAUUCACAGACUUCAUUAAGAUGUGGUGCUUGCAUACGUAGACCAUUCUUAUGUUGUAAA
UGCUGUUACGACCAUGUCAUAUCAACAUCACAUAAAUUAGUCUUGUCUGUUAAUCCGUAU
GUUUGCAAUGCUCCAGGUUGUGAUGUCACAGAUGUGACUCAACUUUACUUAGGAGGUAUG
AGCUAUUAUUGUAAAUCACAUAAACCACCCAUUAGUUUUCCAUUGUGUGCUAAUGGACAA
GUUUUUGGUUUAUAUAAAAAUACAUGUGUUGGUAGCGAUAAUGUUACUGACUUUAAUGCA
AUUGCAACAUGUGACUGGACAAAUGCUGGUGAUUACAUUUUAGCUAACACCUGUACUGAA
AGACUCAAGCUUUUUGCAGCAGAAACGCUCAAAGCUACUGAGGAGACAUUUAAACUGUCU
UAUGGUAUUGCUACUGUACGUGAAGUGCUGUCUGACAGAGAAUUACAUCUUUCAUGGGAA
GUUGGUAAACCUAGACCACCACUUAACCGAAAUUAUGUCUUUACUGGUUAUCGUGUAACU
AAAAACAGUAAAGUACAAAUAGGAGAGUACACCUUUGAAAAAGGUGACUAUGGUGAUGCU
GUUGUUUACCGAGGUACAACAACUUACAAAUUAAAUGUUGGUGAUUAUUUUGUGCUGACA
UCACAUACAGUAAUGCCAUUAAGUGCACCUACACUAGUGCCACAAGAGCACUAUGUUAGA
AUUACUGGCUUAUACCCAACACUCAAUAUCUCAGAUGAGUUUUCUAGCAAUGUUGCAAAU
UAUCAAAAGGUUGGUAUGCAAAAGUAUUCUACACUCCAGGGACCACCUGGUACUGGUAAG
AGUCAUUUUGCUAUUGGCCUAGCUCUCUACUACCCUUCUGCUCGCAUAGUGUAUACAGCU
UGCUCUCAUGCCGCUGUUGAUGCACUAUGUGAGAAGGCAUUAAAAUAUUUGCCUAUAGAU
AAAUGUAGUAGAAUUAUACCUGCACGUGCUCGUGUAGAGUGUUUUGAUAAAUUCAAAGUG
AAUUCAACAUUAGAACAGUAUGUCUUUUGUACUGUAAAUGCAUUGCCUGAGACGACAGCA
GAUAUAGUUGUCUUUGAUGAAAUUUCAAUGGCCACAAAUUAUGAUUUGAGUGUUGUCAAU
GCCAGAUUACGUGCUAAGCACUAUGUGUACAUUGGCGACCCUGCUCAAUUACCUGCACCA
CGCACAUUGCUAACUAAGGGCACACUAGAACCAGAAUAUUUCAAUUCAGUGUGUAGACUU
AUGAAAACUAUAGGUCCAGACAUGUUCCUCGGAACUUGUCGGCGUUGUCCUGCUGAAAUU
GUUGACACUGUGAGUGCUUUGGUUUAUGAUAAUAAGCUUAAAGCACAUAAAGACAAAUCA
GCUCAAUGCUUUAAAAUGUUUUAUAAGGGUGUUAUCACGCAUGAUGUUUCAUCUGCAAUU
AACAGGCCACAAAUAGGCGUGGUAAGAGAAUUCCUUACACGUAACCCUGCUUGGAGAAAA
GCUGUCUUUAUUUCACCUUAUAAUUCACAGAAUGCUGUAGCCUCAAAGAUUUUGGGACUA
CCAACUCAAACUGUUGAUUCAUCACAGGGCUCAGAAUAUGACUAUGUCAUAUUCACUCAA
ACCACUGAAACAGCUCACUCUUGUAAUGUAAACAGAUUUAAUGUUGCUAUUACCAGAGCA
AAAGUAGGCAUACUUUGCAUAAUGUCUGAUAGAGACCUUUAUGACAAGUUGCAAUUUACA
AGUCUUGAAAUUCCACGUAGGAAUGUGGCAACUUUACAAGCUGAAAAUGUAACAGGACUU
UUUAAAGAUUGUAGUAAGGUAAUCACUGGGUUACAUCCUACACAGGCACCUACACACCUC
AGUGUUGACACUAAAUUCAAAACUGAAGGUUUAUGUGUUGACAUACCUGGCAUACCUAAG
GACAUGACCUAUAGAAGACUCAUCUCUAUGAUGGGUUUUAAAAUGAAUUAUCAAGUUAAU
GGUUACCCUAACAUGUUUAUCACCCGCGAAGAAGCUAUAAGACAUGUACGUGCAUGGAUU
GGCUUCGAUGUCGAGGGGUGUCAUGCUACUAGAGAAGCUGUUGGUACCAAUUUACCUUUA
CAGCUAGGUUUUUCUACAGGUGUUAACCUAGUUGCUGUACCUACAGGUUAUGUUGAUACA
CCUAAUAAUACAGAUUUUUCCAGAGUUAGUGCUAAACCACCGCCUGGAGAUCAAUUUAAA
CACCUCAUACCACUUAUGUACAAAGGACUUCCUUGGAAUGUAGUGCGUAUAAAGAUUGUA
CAAAUGUUAAGUGACACACUUAAAAAUCUCUCUGACAGAGUCGUAUUUGUCUUAUGGGCA
CAUGGCUUUGAGUUGACAUCUAUGAAGUAUUUUGUGAAAAUAGGACCUGAGCGCACCUGU
UGUCUAUGUGAUAGACGUGCCACAUGCUUUUCCACUGCUUCAGACACUUAUGCCUGUUGG
CAUCAUUCUAUUGGAUUUGAUUACGUCUAUAAUCCGUUUAUGAUUGAUGUUCAACAAUGG
GGUUUUACAGGUAACCUACAAAGCAACCAUGAUCUGUAUUGUCAAGUCCAUGGUAAUGCA
CAUGUAGCUAGUUGUGAUGCAAUCAUGACUAGGUGUCUAGCUGUCCACGAGUGCUUUGUU
AAGCGUGUUGACUGGACUAUUGAAUAUCCUAUAAUUGGUGAUGAACUGAAGAUUAAUGCG
GCUUGUAGAAAGGUUCAACACAUGGUUGUUAAAGCUGCAUUAUUAGCAGACAAAUUCCCA
GUUCUUCACGACAUUGGUAACCCUAAAGCUAUUAAGUGUGUACCUCAAGCUGAUGUAGAA
UGGAAGUUCUAUGAUGCACAGCCUUGUAGUGACAAAGCUUAUAAAAUAGAAGAAUUAUUC
UAUUCUUAUGCCACACAUUCUGACAAAUUCACAGAUGGUGUAUGCCUAUUUUGGAAUUGC
AAUGUCGAUAGAUAUCCUGCUAAUUCCAUUGUUUGUAGAUUUGACACUAGAGUGCUAUCU
AACCUUAACUUGCCUGGUUGUGAUGGUGGCAGUUUGUAUGUAAAUAAACAUGCAUUCCAC

ACACCAGCUUUUGAUAAAAGUGCUUUUGUUAAUUUAAAACAAUUACCAUUUUUCUAUUAC
UCUGACAGUCCAUGUGAGUCUCAUGGAAAACAAGUAGUGUCAGAUAUAGAUUAUGUACCA
CUAAAGUCUGCUACGUGUAUAACACGUUGCAAUUUAGGUGGUGCUGUCUGUAGACAUCAU
GCUAAUGAGUACAGAUUGUAUCUCGAUGCUUAUAACAUGAUGAUCUCAGCUGGCUUUAGC
UUGUGGGUUUACAAACAAUUUGAUACUUAUAACCUCUGGAACACUUUUACAAGACUUCAG
AGUUUAGAAAAUGUGGCUUUUAAUGUUGUAAAUAAGGGACACUUUGAUGGACAACAGGGU
GAAGUACCAGUUUCUAUCAUUAAUAACACUGUUUACACAAAAGUUGAUGGUGUUGAUGUA
GAAUUGUUUGAAAAUAAAACAACAUUACCUGUUAAUGUAGCAUUUGAGCUUUGGGCUAAG
CGCAACAUUAAACCAGUACCAGAGGUGAAAAUACUCAAUAAUUUGGGUGUGGACAUUGCU
GCUAAUACUGUGAUCUGGGACUACAAAAGAGAUGCUCCAGCACAUAUAUCUACUAUUGGU
GUUUGUUCUAUGACUGACAUAGCCAAGAAACCAACUGAAACGAUUUGUGCACCACUCACU
GUCUUUUUUGAUGGUAGAGUUGAUGGUCAAGUAGACUUAUUUAGAAAUGCCCGUAAUGGU
GUUCUUAUUACAGAAGGUAGUGUUAAAGGUUUACAACCAUCUGUAGGUCCCAAACAAGCU
AGUCUUAAUGGAGUCACAUUAAUUGGAGAAGCCGUAAAAACACAGUUCAAUUAUUAUAAG
AAAGUUGAUGGUGUUGUCCAACAAUUACCUGAAACUUACUUUACUCAGAGUAGAAAUUUA
CAAGAAUUUAAACCCAGGAGUCAAAUGGAAAUUGAUUUCUUAGAAUUAGCUAUGGAUGAA
UUCAUUGAACGGUAUAAAUUAGAAGGCUAUGCCUUCGAACAUAUCGUUUAUGGAGAUUUU
AGUCAUAGUCAGUUAGGUGGUUUACAUCUACUGAUUGGACUAGCUAAACGUUUUAAGGAA
UCACCUUUUGAAUUAGAAGAUUUUAUUCCUAUGGACAGUACAGUUAAAAACUAUUUCAUA
ACAGAUGCGCAAACAGGUUCAUCUAAGUGUGUGUGUUCUGUUAUUGAUUUAUUACUUGAU
GAUUUUGUUGAAAUAAUAAAAUCCCAAGAUUUAUCUGUAGUUUCUAAGGUUGUCAAAGUG
ACUAUUGACUAUACAGAAAUUUCAUUUAUGCUUUGGUGUAAAGAUGGCCAUGUAGAAACA
UUUUACCCAAAAUUACAAUCUAGUCAAGCGUGGCAACCGGGUGUUGCUAUGCCUAAUCUU
UACAAAAUGCAAAGAAUGCUAUUAGAAAAGUGUGACCUUCAAAAUUAUGGUGAUAGUGCA
ACAUUACCUAAAGGCAUAAUGAUGAAUGUCGCAAAAUAUACUCAACUGUGUCAAUAUUUA
AACACAUUAACAUUAGCUGUACCCUAUAAUAUGAGAGUUAUACAUUUUGGUGCUGGUUCU
GAUAAAGGAGUUGCACCAGGUACAGCUGUUUUAAGACAGUGGUUGCCUACGGGUACGCUG
CUUGUCGAUUCAGAUCUUAAUGACUUUGUCUCUGAUGCAGAUUCAACUUUGAUUGGUGAU
UGUGCAACUGUACAUACAGCUAAUAAAUGGGAUCUCAUUAUUAGUGAUAUGUACGACCCU
AAGACUAAAAAUGUUACAAAAGAAAAUGACUCUAAAGAGGGUUUUUUCACUUACAUUUGU
GGGUUUAUACAACAAAAGCUAGCUCUUGGAGGUUCCGUGGCUAUAAAGAUAACAGAACAU
UCUUGGAAUGCUGAUCUUUAUAAGCUCAUGGGACACUUCGCAUGGUGGACAGCCUUUGUU
ACUAAUGUGAAUGCGUCAUCAUCUGAAGCAUUUUUAAUUGGAUGUAAUUAUCUUGGCAAA
CCACGCGAACAAAUAGAUGGUUAUGUCAUGCAUGCAAAUUACAUAUUUUGGAGGAAUACA
AAUCCAAUUCAGUUGUCUUCCUAUUCUUUAUUUGACAUGAGUAAAUUUCCCCUUAAAUUA
AGGGGUACUGCUGUUAUGUCUUUAAAAGAAGGUCAAAUCAAUGAUAUGAUUUUAUCUCUU
CUUAGUAAAGGUAGACUUAUAAUUAGAGAAAACAACAGAGUUGUUAUUUCUAGUGAUGUU
CUUGUUAACAACUAAACGAACAAUGUUUGUUUUUCUUGUUUUAUUGCCACUAGUCUCUAG
UCAGUGUGUUAAUCUUACAACCAGAACUCAAUUACCCCCUGCAUACACUAAUUCUUUCAC
ACGUGGUGUUUAUUACCCUGACAAAGUUUUCAGAUCCUCAGUUUUACAUUCAACUCAGGA
CUUGUUCUUACCUUUCUUUUCCAAUGUUACUUGGUUCCAUGCUAUACAUGUCUCUGGGAC
CAAUGGUACUAAGAGGUUUGAUAACCCUGUCCUACCAUUUAAUGAUGGUGUUUAUUUUGC
UUCCACUGAGAAGUCUAACAUAAUAAGAGGCUGGAUUUUUGGUACUACUUUAGAUUCGAA
GACCCAGUCCCUACUUAUUGUUAAUAACGCUACUAAUGUUGUUAUUAAAGUCUGUGAAUU
UCAAUUUUGUAAUGAUCCAUUUUUGGGUGUUUAUUACCACAAAAACAACAAAAGUUGGAU
GGAAAGUGAGUUCAGAGUUUAUUCUAGUGCGAAUAAUUGCACUUUUGAAUAUGUCUCUCA
GCCUUUUCUUAUGGACCUUGAAGGAAAACAGGGUAAUUUCAAAAAUCUUAGGGAAUUUGU
GUUUAAGAAUAUUGAUGGUUAUUUUAAAAUAUAUUCUAAGCACACGCCUAUUAAUUUAGU
GCGUGAUCUCCCUCAGGGUUUUUCGGCUUUAGAACCAUUGGUAGAUUUGCCAAUAGGUAU
UAACAUCACUAGGUUUCAAACUUUACUUGCUUUACAUAGAAGUUAUUUGACUCCUGGUGA
UUCUUCUUCAGGUUGGACAGCUGGUGCUGCAGCUUAUUAUGUGGGUUAUCUUCAACCUAG
GACUUUUCUAUUAAAAUAUAAUGAAAAUGGAACCAUUACAGAUGCUGUAGACUGUGCACU
UGACCCUCUCUCAGAAACAAAGUGUACGUUGAAAUCCUUCACUGUAGAAAAAGGAAUCUA
UCAAACUUCUAACUUUAGAGUCCAACCAACAGAAUCUAUUGUUAGAUUUCCUAAUAUUAC
AAACUUGUGCCCUUUUGGUGAAGUUUUUAACGCCACCAGAUUUGCAUCUGUUUAUGCUUG
GAACAGGAAGAGAAUCAGCAACUGUGUUGCUGAUUAUUCUGUCCUAUAUAAUUCCGCAUC
AUUUUCCACUUUUAAGUGUUAUGGAGUGUCUCCUACUAAAUUAAAUGAUCUCUGCUUUAC
UAAUGUCUAUGCAGAUUCAUUUGUAAUUAGAGGUGAUGAAGUCAGACAAAUCGCUCCAGG
GCAAACUGGAAAGAUUGCUGAUUAUAAUUAUAAAUUACCAGAUGAUUUUACAGGCUGCGU
UAUAGCUUGGAAUUCUAACAAUCUUGAUUCUAAGGUUGGUGGUAAUUAUAAUUACCUGUA
UAGAUUGUUUAGGAAGUCUAAUCUCAAACCUUUUGAGAGAGAUAUUUCAACUGAAAUCUA
UCAGGCCGGUAGCACACCUUGUAAUGGUGUUGAAGGUUUUAAUUGUUACUUUCCUUUACA
AUCAUAUGGUUUCCAACCCACUAAUGGUGUUGGUUACCAACCAUACAGAGUAGUAGUACU
UUCUUUUGAACUUCUACAUGCACCAGCAACUGUUUGUGGACCUAAAAAGUCUACUAAUUU
GGUUAAAAACAAAUGUGUCAAUUUCAACUUCAAUGGUUUAACAGGCACAGGUGUUCUUAC
UGAGUCUAACAAAAAGUUUCUGCCUUUCCAACAAUUUGGCAGAGACAUUGCUGACACUAC
UGAUGCUGUCCGUGAUCCACAGACACUUGAGAUUCUUGACAUUACACCAUGUUCUUUUGG
UGGUGUCAGUGUUAUAACACCAGGAACAAAUACUUCUAACCAGGUUGCUGUUCUUUAUCA

GGAUGUUAACUGCACAGAAGUCCCUGUUGCUAUUCAUGCAGAUCAACUUACUCCUACUUG
GCGUGUUUAUUCUACAGGUUCUAAUGUUUUUCAAACACGUGCAGGCUGUUUAAUAGGGGC
UGAACAUGUCAACAACUCAUAUGAGUGUGACAUACCCAUUGGUGCAGGUAUAUGCGCUAG
UUAUCAGACUCAGACUAAUUCUCCUCGGCGGGCACGUAGUGUAGCUAGUCAAUCCAUCAU
UGCCUACACUAUGUCACUUGGUGCAGAAAAUUCAGUUGCUUACUCUAAUAACUCUAUUGC
CAUACCCACAAAUUUUACUAUUAGUGUUACCACAGAAAUUCUACCAGUGUCUAUGACCAA
GACAUCAGUAGAUUGUACAAUGUACAUUUGUGGUGAUUCAACUGAAUGCAGCAAUCUUUU
GUUGCAAUAUGGCAGUUUUUGUACACAAUUAAACCGUGCUUUAACUGGAAUAGCUGUUGA
ACAAGACAAAAACACCCAAGAAGUUUUUGCACAAGUCAAACAAAUUUACAAAACACCACC
AAUUAAAGAUUUUGGUGGUUUUAAUUUUUCACAAAUAUUACCAGAUCCAUCAAAACCAAG
CAAGAGGUCAUUUAUUGAAGAUCUACUUUUCAACAAAGUGACACUUGCAGAUGCUGGCUU
CAUCAAACAAUAUGGUGAUUGCCUUGGUGAUAUUGCUGCUAGAGACCUCAUUUGUGCACA
AAAGUUUAACGGCCUUACUGUUUUGCCACCUUUGCUCACAGAUGAAAUGAUUGCUCAAUA
CACUUCUGCACUGUUAGCGGGUACAAUCACUUCUGGUUGGACCUUUGGUGCAGGUGCUGC
AUUACAAAUACCAUUUGCUAUGCAAAUGGCUUAUAGGUUUAAUGGUAUUGGAGUUACACA
GAAUGUUCUCUAUGAGAACCAAAAAUUGAUUGCCAACCAAUUUAAUAGUGCUAUUGGCAA
AAUUCAAGACUCACUUUCUUCCACAGCAAGUGCACUUGGAAAACUUCAAGAUGUGGUCAA
CCAAAAUGCACAAGCUUUAAACACGCUUGUUAAACAACUUAGCUCCAAUUUUGGUGCAAU
UUCAAGUGUUUUAAAUGAUAUCCUUUCACGUCUUGACAAAGUUGAGGCUGAAGUGCAAAU
UGAUAGGUUGAUCACAGGCAGACUUCAAAGUUUGCAGACAUAUGUGACUCAACAAUUAAU
UAGAGCUGCAGAAAUCAGAGCUUCUGCUAAUCUUGCUGCUACUAAAAUGUCAGAGUGUGU
ACUUGGACAAUCAAAAAGAGUUGAUUUUUGUGGAAAGGGCUAUCAUCUUAUGUCCUUCCC
UCAGUCAGCACCUCAUGGUGUAGUCUUCUUGCAUGUGACUUAUGUCCCUGCACAAGAAAA
GAACUUCACAACUGCUCCUGCCAUUUGUCAUGAUGGAAAAGCACACUUUCCUCGUGAAGG
UGUCUUUGUUUCAAAUGGCACACACUGGUUUGUAACACAAAGGAAUUUUUAUGAACCACA
AAUCAUUACUACAGACAACACAUUUGUGUCUGGUAACUGUGAUGUUGUAAUAGGAAUUGU
CAACAACACAGUUUAUGAUCCUUUGCAACCUGAAUUAGACUCAUUCAAGGAGGAGUUAGA
UAAAUAUUUUAAGAAUCAUACAUCACCAGAUGUUGAUUUAGGUGACAUCUCUGGCAUUAA
UGCUUCAGUUGUAAACAUUCAAAAAGAAAUUGACCGCCUCAAUGAGGUUGCCAAGAAUUU
AAAUGAAUCUCUCAUCGAUCUCCAAGAACUUGGAAAGUAUGAGCAGUAUAUAAAAUGGCC
AUGGUACAUUUGGCUAGGUUUUAUAGCUGGCUUGAUUGCCAUAGUAAUGGUGACAAUUAU
GCUUUGCUGUAUGACCAGUUGCUGUAGUUGUCUCAAGGGCUGUUGUUCUUGUGGAUCCUG
CUGCAAAUUUGAUGAAGACGACUCUGAGCCAGUGCUCAAAGGAGUCAAAUUACAUUACAC
AUAAACGAACUUAUGGAUUUGUUUAUGAGAAUCUUCACAAUUGGAACUGUAACUUUGAAG
CAAGGUGAAAUCAAGGAUGCUACUCCUUCAGAUUUUGUUCGCGCUACUGCAACGAUACCG
AUACAAGCCUCACUCCCUUUCGGAUGGCUUAUUGUUGGCGUUGCACUUCUUGCUGUUUUU
CAGAGCGCUUCCAAAAUCAUAACCCUCAAAAAGAGAUGGCAACUAGCACUCUCCAAGGGU
GUUCACUUUGUUUGCAACUUGCUGUUGUUGUUUGUAACAGUUUACUCACACCUUUUGCUC
GUUGCUGCUGGCCUUGAAGCCCCUUUUCUCUAUCUUUAUGCUUUAGUCUACUUCUUGCAG
AGUAUAAACUUUGUAAGAAUAAUAAUGAGGCUUUGGCUUUGCUGGAAAUGCCGUUCCAAA
AACCCAUUACUUUAUGAUGCCAACUAUUUUCUUUGCUGGCAUACUAAUUGUUACGACUAU
UGUAUACCUUACAAUAGUGUAACUUCUUCAAUUGUCAUUACUUCAGGUGAUGGCACAACA
AGUCCUAUUUCUGAACAUGACUACCAGAUUGGUGGUUAUACUGAAAAAUGGGAAUCUGGA
GUAAAAGACUGUGUUGUAUUACACAGUUACUUCACUUCAGACUAUUACCAGCUGUACUCA
ACUCAAUUGAGUACAGACACUGGUGUUGAACAUGUUACCUUCUUCAUCUACAAUAAAAUU
GUUGAUGAGCCUGAAGAACAUGUCCAAAUUCACACAAUCGACGGUUCAUCCGGAGUUGUU
AAUCCAGUAAUGGAACCAAUUUAUGAUGAACCGACGACGACUACUAGCGUGCCUUUGUAA
GCACAAGCUGAUGAGUACGAACUUAUGUACUCAUUCGUUUCGGAAGAGACAGGUACGUUA
AUAGUUAAUAGCGUACUUCUUUUUCUUGCUUUCGUGGUAUUCUUGCUAGUUACACUAGCC
AUCCUUACUGCGCUUCGAUUGUGUGCGUACUGCUGCAAUAUUGUUAACGUGAGUCUUGUA
AAACCUUCUUUUUACGUUUACUCUCGUGUUAAAAAUCUGAAUUCUUCUAGAGUUCCUGAU
CUUCUGGUCUAAACGAACUAAAUAUUAUAUUAGUUUUUCUGUUUGGAACUUUAAUUUUAG
CCAUGGCAGAUUCCAACGGUACUAUUACCGUUGAAGAGCUUAAAAAGCUCCUUGAACAAU
GGAACCUAGUAAUAGGUUUCCUAUUCCUUACAUGGAUUUGUCUUCUACAAUUUGCCUAUG
CCAACAGGAAUAGGUUUUUGUAUAUAAUUAAGUUAAUUUUCCUCUGGCUGUUAUGGCCAG
UAACUUUAGCUUGUUUUGUGCUUGCUGCUGUUUACAGAAUAAAUUGGAUCACCGGUGGAA
UUGCUAUCGCAAUGGCUUGUCUUGUAGGCUUGAUGUGGCUCAGCUACUUCAUUGCUUCUU
UCAGACUGUUUGCGCGUACGCGUUCCAUGUGGUCAUUCAAUCCAGAAACUAACAUUCUUC
UCAACGUGCCACUCCAUGGCACUAUUCUGACCAGACCGCUUCUAGAAAGUGAACUCGUAA
UCGGAGCUGUGAUCCUUCGUGGACAUCUUCGUAUUGCUGGACACCAUCUAGGACGCUGUG
ACAUCAAGGACCUGCCUAAAGAAAUCACUGUUGCUACAUCACGAACGCUUUCUUAUUACA
AAUUGGGAGCUUCGCAGCGUGUAGCAGGUGACUCAGGUUUUGCUGCAUACAGUCGCUACA
GGAUUGGCAACUAUAAAUUAAACACAGACCAUUCCAGUAGCAGUGACAAUAUUGCUUUGC
UUGUACAGUAAGUGACAACAGAUGUUUCAUCUCGUUGACUUUCAGGUUACUAUAGCAGAG
AUAUUACUAAUUAUUAUGAGGACUUUUAAAGUUUCCAUUUGGAAUCUUGAUUACAUCAUA
AACCUCAUAAUUAAAAAUUUAUCUAAGUCACUAACUGAGAAUAAAUAUUCUCAAUUAGAU
GAAGAGCAACCAAUGGAGAUUGAUUAAACGAACAUGAAAAUUAUUCUUUUCUUGGCACUG
AUAACACUCGCUACUUGUGAGCUUUAUCACUACCAAGAGUGUGUUAGAGGUACAACAGUA

CUUUUAAAAGAACCUUGCUCUUCUGGAACAUACGAGGGCAAUUCACCAUUUCAUCCUCUA
GCUGAUAACAAAUUUGCACUGACUUGCUUUAGCACUCAAUUUGCUUUUGCUUGUCCUGAC
GGCGUAAAACACGUCUAUCAGUUACGUGCCAGAUCAGUUUCACCUAAACUGUUCAUCAGA
CAAGAGGAAGUUCAAGAACUUUACUCUCCAAUUUUUCUUAUUGUUGCGGCAAUAGUGUUU
AUAACACUUUGCUUCACACUCAAAAGAAAGACAGAAUGAUUGAACUUUCAUUAAUUGACU
UCUAUUUGUGCUUUUUAGCCUUUCUGCUAUUCCUUGUUUUAAUUAUGCUUAUUAUCUUUU
GGUUCUCACUUGAACUGCAAGAUCAUAAUGAAACUUGUCACGCCUAAACGAACAUGAAAU
UUCUUGUUUUCUUAGGAAUCAUCACAACUGUAGCUGCAUUUCACCAAGAAUGUAGUUUAC
AGUCAUGUACUCAACAUCAACCAUAUGUAGUUGAUGACCCGUGUCCUAUUCACUUCUAUU
CUAAAUGGUAUAUUAGAGUAGGAGCUAGAAAAUCAGCACCUUUAAUUGAAUUGUGCGUGG
AUGAGGCUGGUUCUAAAUCACCCAUUCAGUACAUCGAUAUCGGUAAUUAUACAGUUUCCU
GUUCACCUUUUACAAUUAAUUGCCAGGAACCUAAAUUGGGUAGUCUUGUAGUGCGUUGUU
CGUUCUAUGAAGACUUUUUAGAGUAUCAUGACGUUCGUGUUGUUUUAGAUUUCAUCUAAA
CGAACAAACUAAAAUGUCUGAUAAUGGACCCCAAAAUCAGCGAAAUGCACCCCGCAUUAC
GUUUGGUGGACCCUCAGAUUCAACUGGCAGUAACCAGAAUGGAGAACGCAGUGGGGCGCG
AUCAAAACAACGUCGGCCCCAAGGUUUACCCAAUAAUACUGCGUCUUGGUUCACCGCUCU
CACUCAACAUGGCAAGGAAGACCUUAAAUUCCCUCGAGGACAAGGCGUUCCAAUUAACAC
CAAUAGCAGUCCAGAUGACCAAAUUGGCUACUACCGAAGAGCUACCAGACGAAUUCGUGG
UGGUGACGGUAAAAUGAAAGAUCUCAGUCCAAGAUGGUAUUUCUACUACCUAGGAACUGG
GCCAGAAGCUGGACUUCCCUAUGGUGCUAACAAAGACGGCAUCAUAUGGGUUGCAACUGA
GGGAGCCUUGAAUACACCAAAAGAUCACAUUGGCACCCGCAAUCCUGCUAACAAUGCUGC
AAUCGUGCUACAACUUCCUCAAGGAACAACAUUGCCAAAAGGCUUCUACGCAGAAGGGAG
CAGAGGCGGCAGUCAAGCCUCUUCUCGUUCCUCAUCACGUAGUCGCAACAGUUCAAGAAA
UUCAACUCCAGGCAGCAGUAGGGGAACUUCUCCUGCUAGAAUGGCUGGCAAUGGCGGUGA
UGCUGCUCUUGCUUUGCUGCUGCUUGACAGAUUGAACCAGCUUGAGAGCAAAAUGUCUGG
UAAAGGCCAACAACAACAAGGCCAAACUGUCACUAAGAAAUCUGCUGCUGAGGCUUCUAA
GAAGCCUCGGCAAAAACGUACUGCCACUAAAGCAUACAAUGUAACACAAGCUUUCGGCAG
ACGUGGUCCAGAACAAACCCAAGGAAAUUUUGGGGACCAGGAACUAAUCAGACAAGGAAC
UGAUUACAAACAUUGGCCGCAAAUUGCACAAUUUGCCCCCAGCGCUUCAGCGUUCUUCGG
AAUGUCGCGCAUUGGCAUGGAAGUCACACCUUCGGGAACGUGGUUGACCUACACAGGUGC
CAUCAAAUUGGAUGACAAAGAUCCAAAUUUCAAAGAUCAAGUCAUUUUGCUGAAUAAGCA
UAUUGACGCAUACAAAACAUUCCCACCAACAGAGCCUAAAAAGGACAAAAAGAAGAAGGC
UGAUGAAACUCAAGCCUUACCGCAGAGACAGAAGAAACAGCAAACUGUGACUCUUCUUCC
UGCUGCAGAUUUGGAUGAUUUCUCCAAACAAUUGCAACAAUCCAUGAGCAGUGCUGACUC
AACUCAGGCCUAAACUCAUGCAGACCACACAAGGCAGAUGGGCUAUAUAAACGUUUUCGC
UUUUCCGUUUACGAUAUAUAGUCUACUCUUGUGCAGAAUGAAUUCUCGUAACUACAUAGC
ACAAGUAGAUGUAGUUAACUUUAAUCUCACAUAGCAAUCUUUAAUCAGUGUGUAACAUUA
GGGAGGACUUGAAAGAGCCACCACAUUUUCACCGAGGCCACGCGGAGUACGAUCGAGUGU
ACAGUGAACAAUGCUAGGGAGAGCUGCCUAUAUGGAAGAGCCCUAAUGUGUAAAAUUAAU
UUUAGUAGUGCUAUCCCCAUGUGAUUUUAAUAGCUUCUUAGGAGAAUGACAAAAAAAAAA
AA

Interaction probabilities

Prediction using RF classifier 0.8
Prediction using SVM classifier 1

What do these probabilities mean?
Interaction probabilities generated by RPISeq range from 0 to 1. In performance evaluation experiments, predictions with probabilities > 0.5 were considered “positive,” i.e., indicating that the corresponding RNA and protein are likely to interact. Using this threshold, accuracies of the classifiers ranged from 87 - 90% in cross-validation evaluation experiments on benchmark datasets. When classifiers were tested on independent (blind) datasets of RPIs, accuracies of the classifiers ranged from 57 – 99%.

Please see About/FAQs for additional details.

Dobbs Lab, (515) 294 4991, usha (at) iastate.edu
Copyright © 2011 Dobbs Lab. All rights reserved.
