# Supplementary figures and images for: Nuclear translocation of spike mRNA and protein is a novel feature of SARS-CoV-2
Source: Front Microbiol. 2023 Jan 26;14:1073789. doi: 10.3389/fmicb.2023.1073789 (PMC9909199; doi:10.3389/fmicb.2023.1073789)

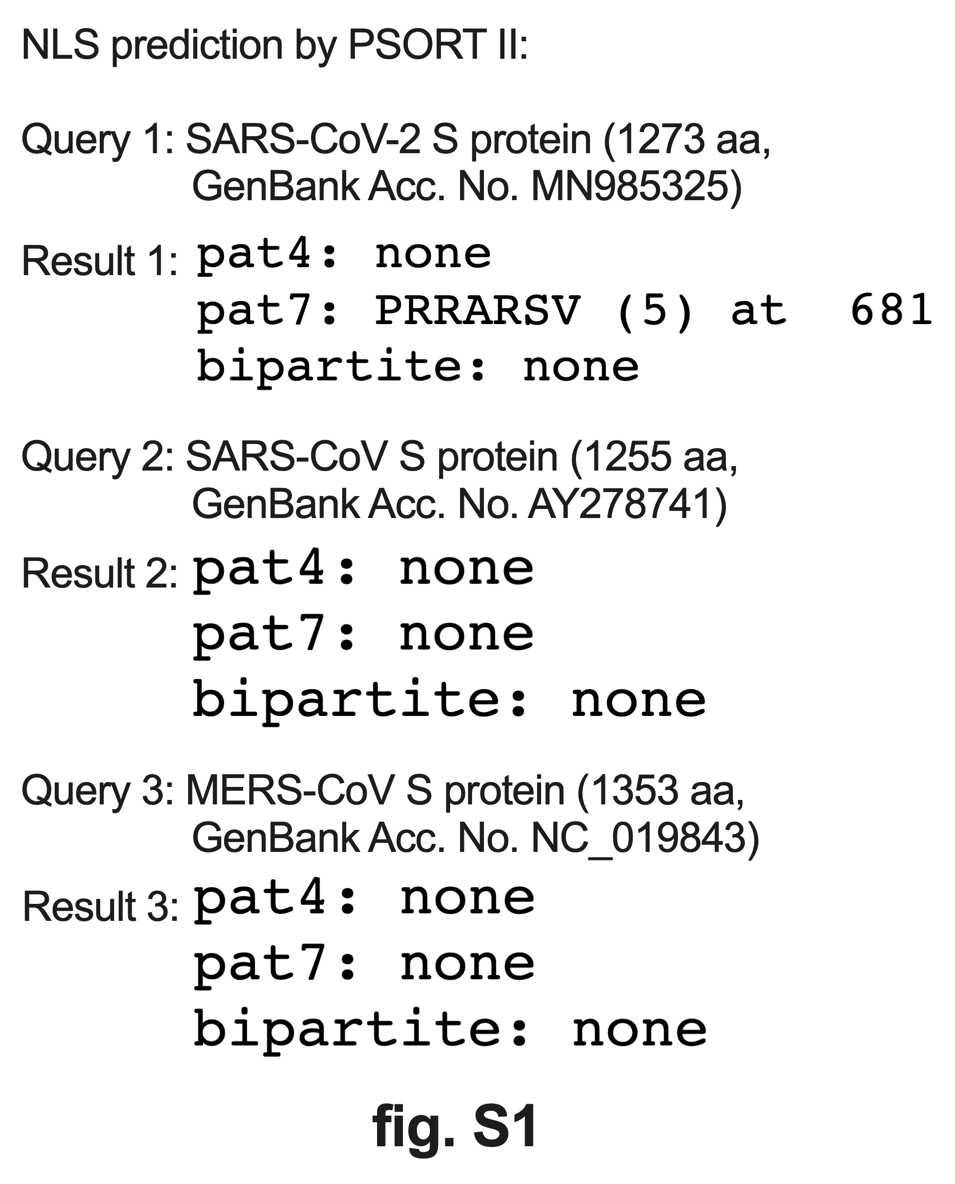

Supplement: Supplementary file 3 [file Image_1.TIFF]

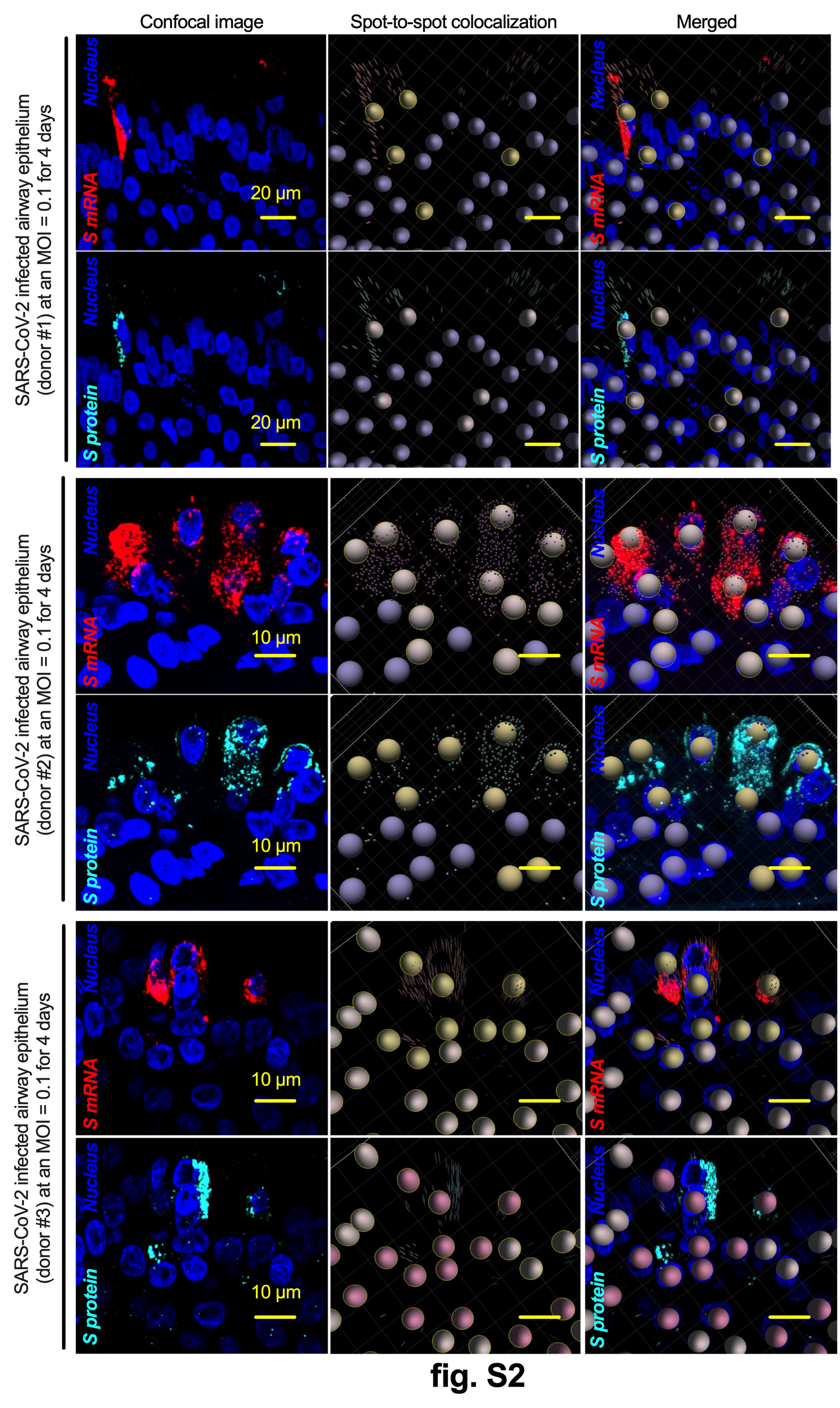

Supplement: Supplementary file 4 [file Image_2.TIFF]

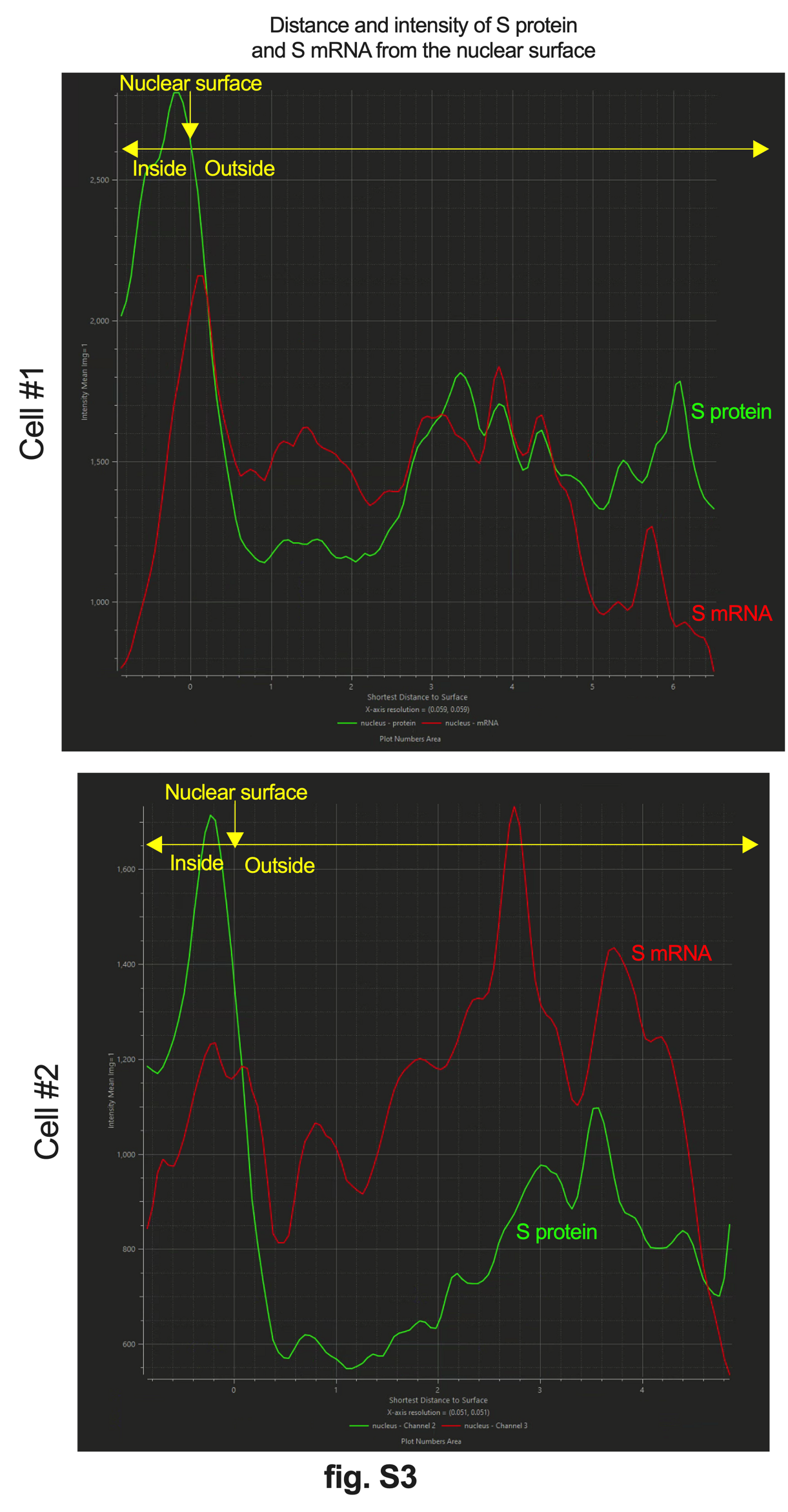

Supplement: Supplementary file 5 [file Image_3.TIFF]

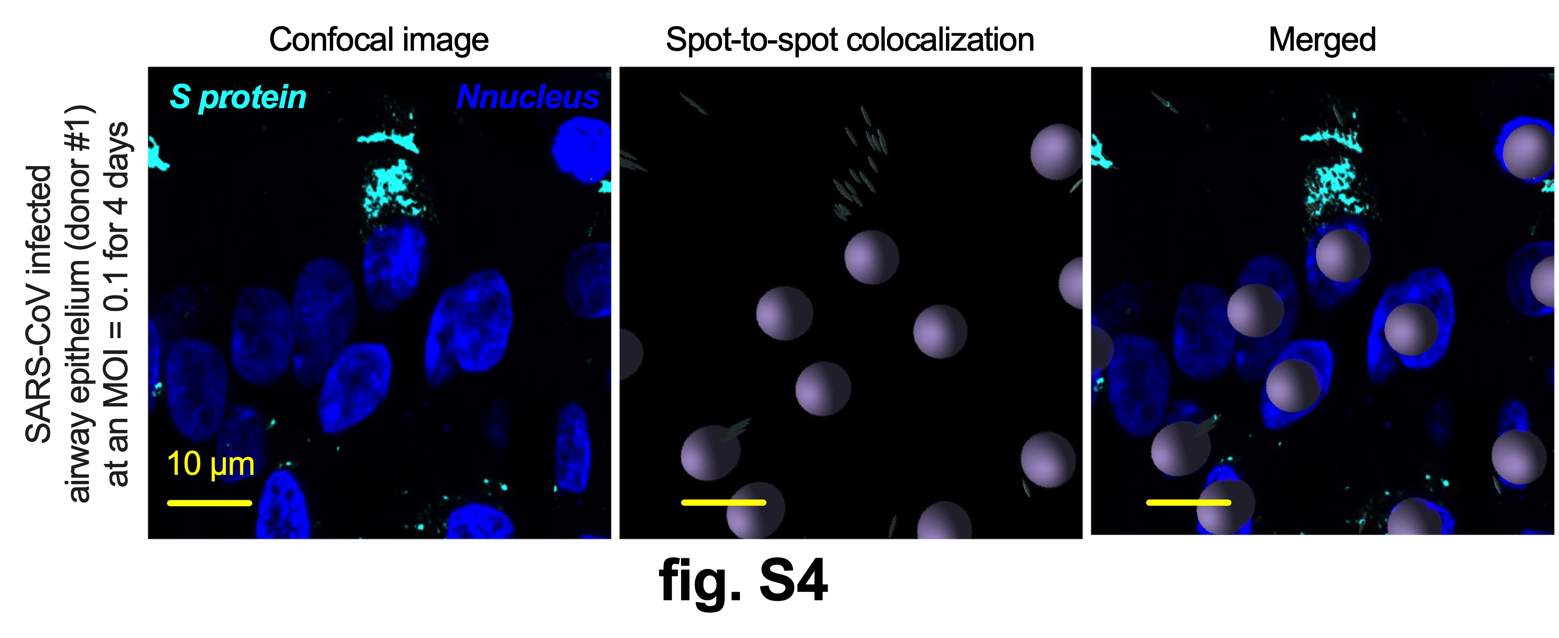

Supplement: Supplementary file 6 [file Image_4.TIFF]

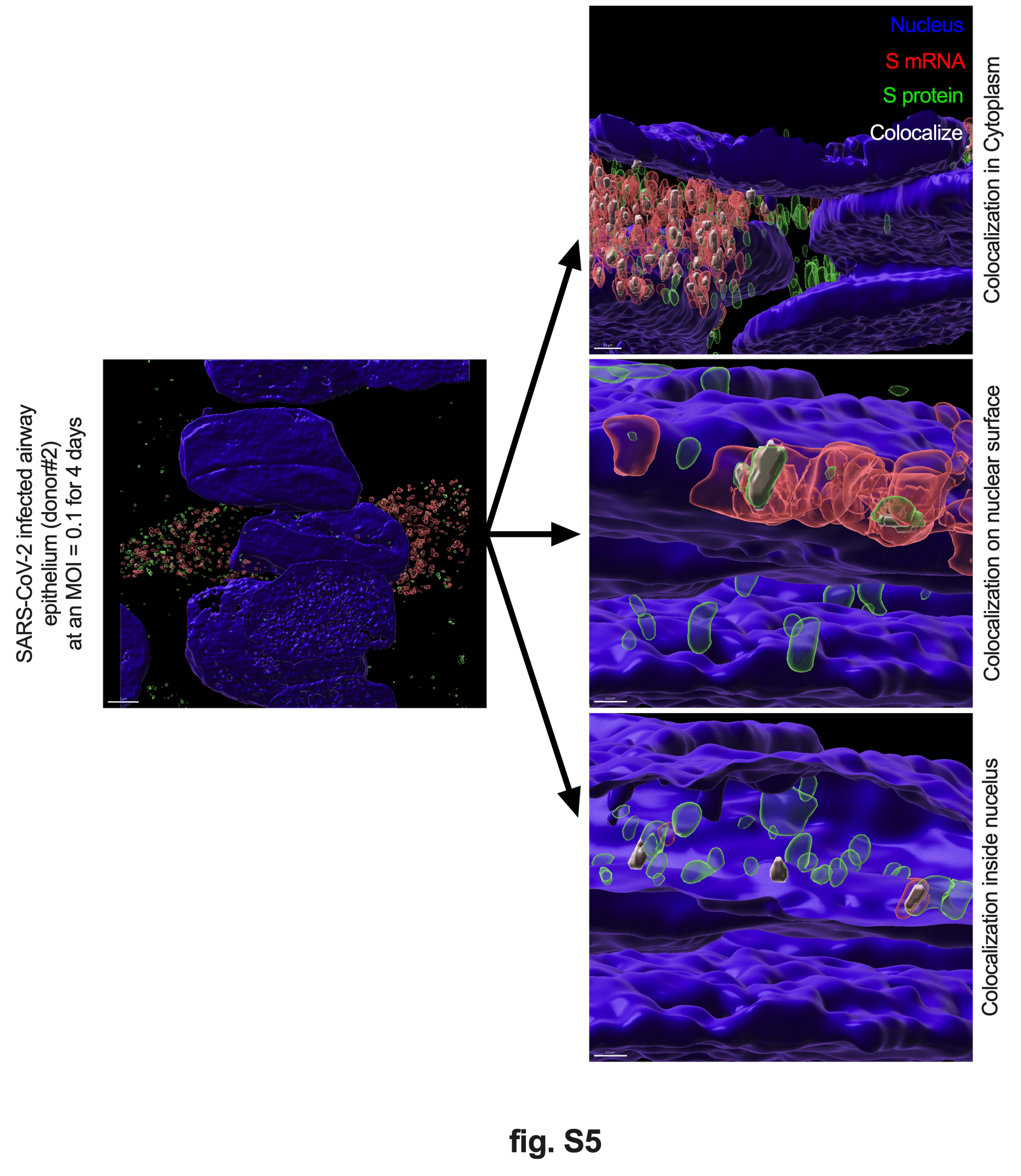

Supplement: Supplementary file 7 [file Image_5.TIFF]

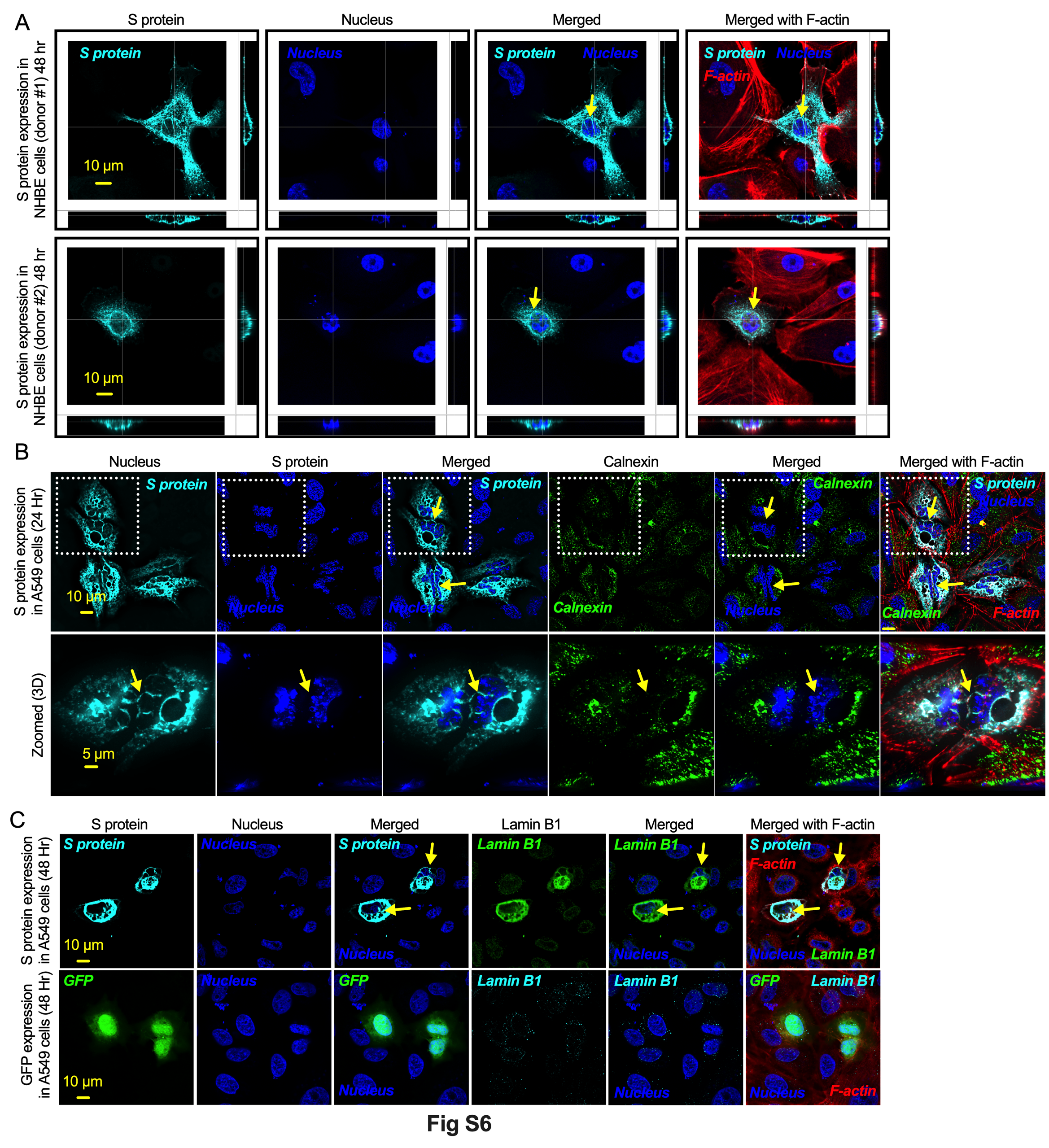

Supplement: Supplementary file 8 [file Image_6.TIFF]

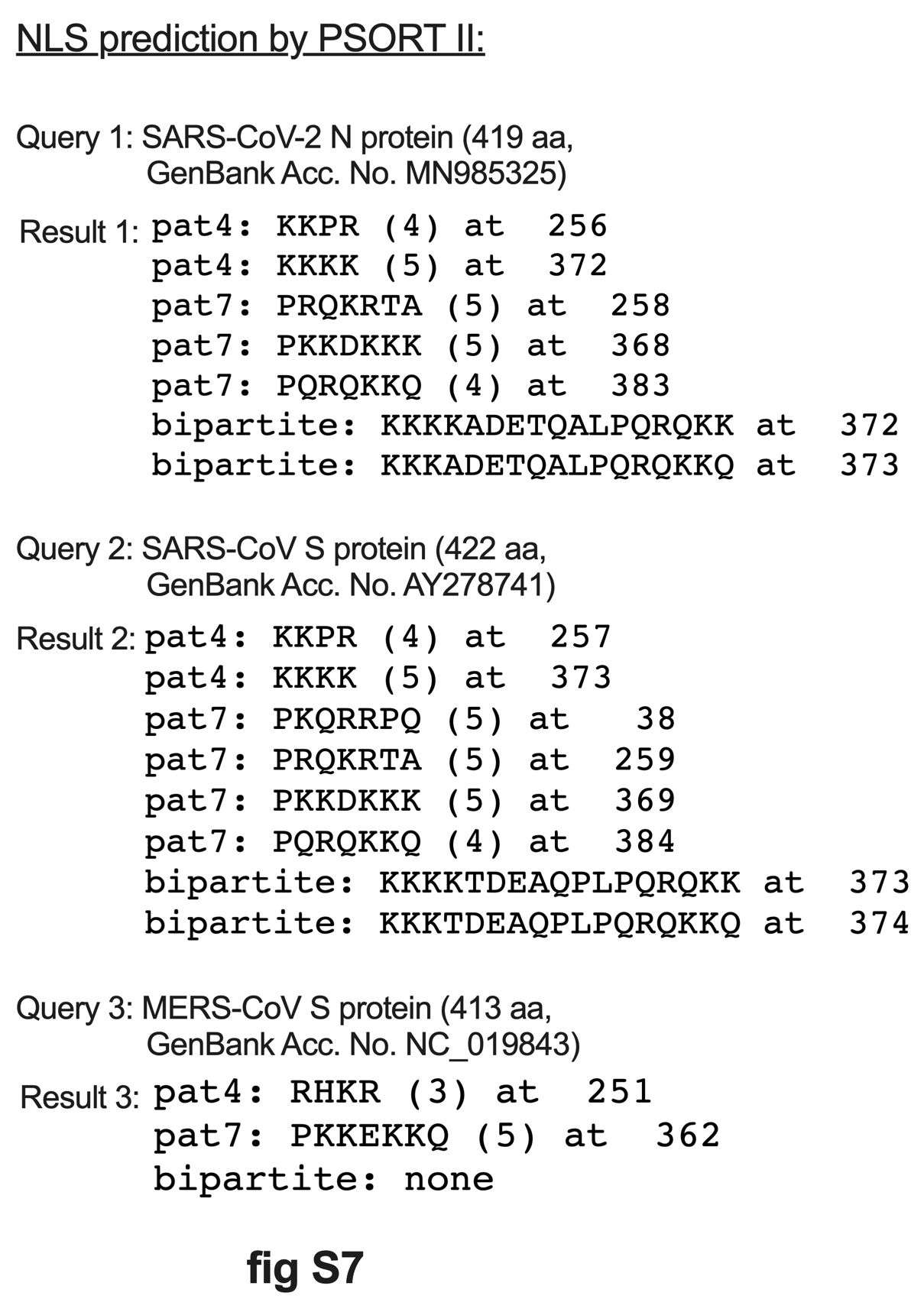

Supplement: Supplementary file 9 [file Image_7.TIFF]

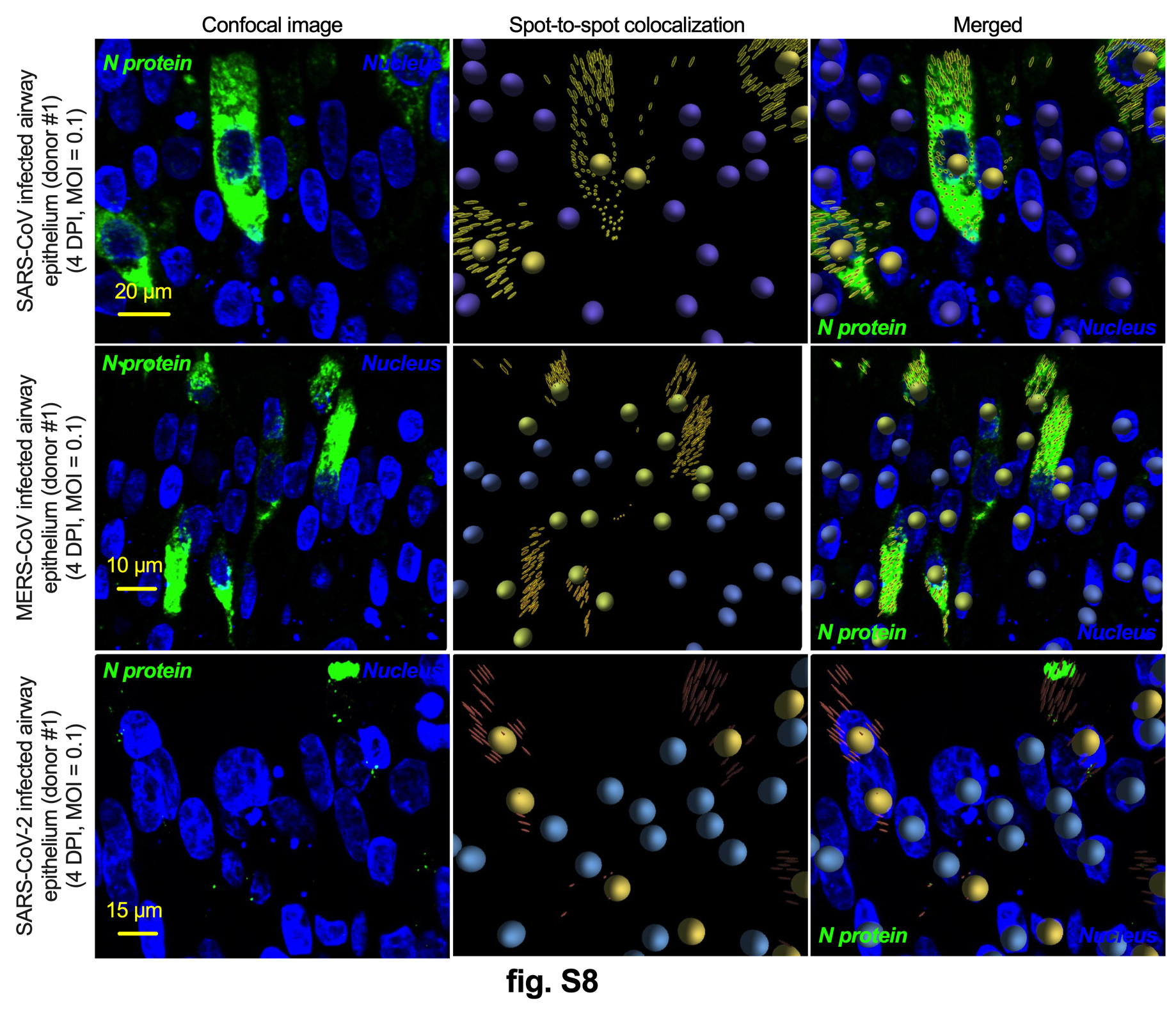

Supplement: Supplementary file 10 [file Image_8.TIFF]

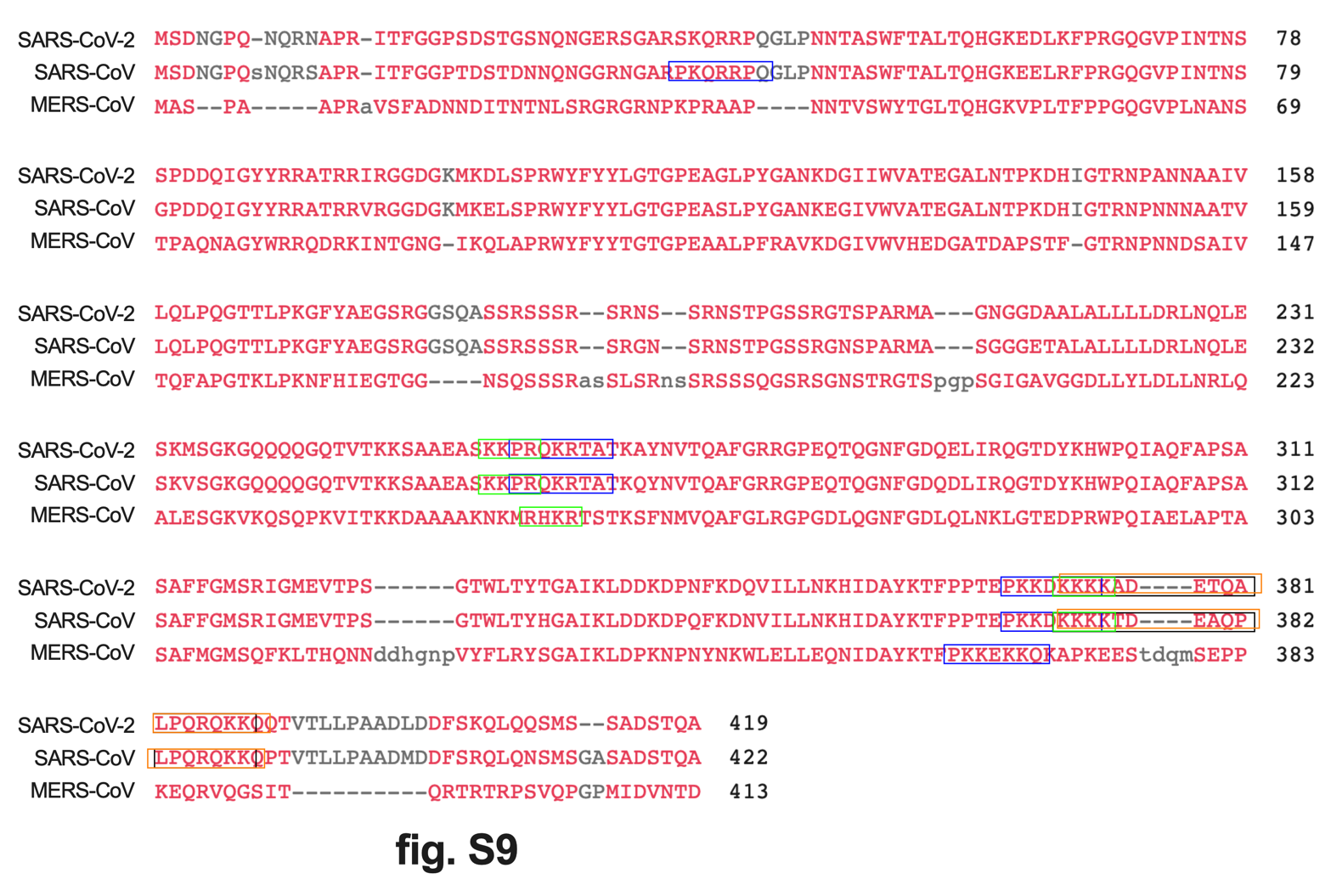

Supplement: Supplementary file 11 [file Image_9.TIFF]
